# Evaluation of Polymeric Micro/Nanofibrous Hybrid Scaffolds Prepared via Centrifugal Nozzleless Spinning for Tissue Engineering Applications

**DOI:** 10.3390/polym17030386

**Published:** 2025-01-31

**Authors:** Miloš Beran, Jana Musílková, Antonín Sedlář, Petr Slepička, Martin Veselý, Zdeňka Kolská, Ondřej Vltavský, Martin Molitor, Lucie Bačáková

**Affiliations:** 1 Czech Agrifood Research Center, Drnovská 73, 161 00 Prague, Czech Republic; 2 Institute of Physiology of the Czech Academy of Sciences, Videnska 1083, 142 00 Prague, Czech Republic; 3 Department of Solid State Engineering, University of Chemistry and Technology Prague, Technicka 5, 166 28 Prague, Czech Republic; 4 Department of Organic Technology, University of Chemistry and Technology Prague, Technicka 5, 166 28 Prague, Czech Republic; 5 J. E. Purkyne University in Usti nad Labem, Pasteurova 3544/1, 400 96 Usti nad Labem, Czech Republic; 6 Department of Plastic Surgery, First Faculty of Medicine, Charles University, Na Bulovce Hospital, Budinova 67/2, 180 81 Prague, Czech Republic

**Keywords:** micro/nanofibrous scaffolds, centrifugal spinning technology, tissue engineering, PLA, PHB, PCL, PBS, bioartificial adipose tissue, hierarchical inner porosity of fibers, mesenchymal stem cells

## Abstract

We compared the applicability of 3D fibrous scaffolds, produced by our patented centrifugal spinning technology, in soft tissue engineering. The scaffolds were prepared from four different biocompatible and biodegradable thermoplastics, namely, polylactide (PLA), polycaprolactone (PCL), poly(3-hydroxybutyrate) (PHB), and poly(1,4-butylene succinate) (PBS) and their blends. The combined results of SEM and BET analyses revealed an internal hierarchically organized porosity of the polymeric micro/nanofibers. Both nanoporosity and capillary effect are crucial for the water retention capacity of scaffolds designed for tissue engineering. The increased surface area provided by nanoporosity enhances water retention, while the capillary effect facilitates the movement of water and nutrients within the scaffolds. When the scaffolds were seeded with adipose-derived stem cells (ASCs), the ingrowth of these cells was the deepest in the PLA/PCL 13.5/4 (*w*/*w*) composite scaffolds. This result is consistent with the relatively large pore size in the fibrous networks, the high internal porosity, and the large specific surface area found in these scaffolds, which may therefore be best suited as a component of adipose tissue substitutes that could reduce postoperative tissue atrophy. Adipose tissue constructs produced in this way could be used in the future instead of conventional fat grafts, for example, in breast reconstruction following cancer ablation.

## 1. Introduction

Tissue engineering combines isolated functional cells and a biodegradable biomaterial to promote the regeneration or repair of diseased or injured tissues. A highly porous scaffold plays a critical role in accommodating cells and guiding their growth and tissue regeneration in three dimensions. Nanofibrous scaffolds are considered to be one of the most suitable scaffolds for tissue engineering because they mimic the nanofibrous architecture of the natural extracellular matrix (ECM) (for a review, see [1]). Currently, a variety of techniques are available to create nanofibers, such as electrospinning (DC, AC), centrifugal spinning, centrifugal electrospinning, solution blow spinning, self-assembly, melt spinning, phase separation, or solid-free form fabrication [2]. Recently, modified techniques of reactive electrospinning has been reviewed [3]. A relatively new approach for fabricating in situ or post-crosslinked nanofibers allows us to improve the physicochemical and mechanical properties of the fabricated nanofibers, making them more biocompatible and tailored for advanced smart drug delivery and tissue engineering applications.

The availability of a wide range of natural and synthetic biomaterials has widened the scope for the development of nanofibrous scaffolds. The three-dimensional (3D) synthetic biodegradable scaffolds designed using nanofibers serve as an excellent substrate for cell adhesion, proliferation, and differentiation. Therefore, nanofibers, irrespective of their method of fabrications, have been used as scaffolds for musculoskeletal tissue engineering (including bone, cartilage, ligament, and skeletal muscle), skin tissue engineering, vascular tissue engineering, neural tissue engineering, and as carriers for the controlled delivery of drugs, proteins, and DNA [4,5]. Tissue engineering uses a combination of cell biology, chemistry, and biomaterials to create 3D tissues that mimic the architecture of the native ECM, consisting of diverse interwoven nanofibrous structures. The unique properties of polymeric nanofibers make them a valuable tool to tissue engineers. In this field, the term “nanofiber” is typically used to describe fibers with diameters ranging from 1 to 1000 nm.

The small diameter of nanofibers closely matches the size scale of ECM fibers, allowing them to be used as biomimetic scaffolds, and the high surface area-to-volume ratio is ideal for cell attachment and drug loading. Compared to macroscale surfaces, nanofibers have shown higher rates of protein adsorption, a key mediator in cell attachment to a biomaterial surface. In addition, polymeric nanofibers have been shown to exhibit unique mechanical properties. In particular, tensile modulus, tensile strength, and shear modulus have been shown to increase with decreasing fiber diameter [6]. Sustainable nanofibers in tissue engineering and biomedical applications, methods for their preparation [2], and nanofiber applications for wound dressing and tissue engineering [7] have recently been reviewed. Polymeric nanofibers can also be embedded in 3D-printed hydrogels to enhance cell–material interactions in biomedical applications. As a biomimetic scaffold, the nanofiber-reinforced hydrogels showed improved compressive modulus and cell growth [8].

The most widely used method for producing nanofibrous scaffolds is electrospinning. Most other nanofiber fabrication methods, such as melt-blowing, bicomponent fiber spinning, phase separation, template synthesis, and self-assembly, are complex and can only be used to produce nanofibers from a limited range of polymers. The production of nanofibrous scaffolds via the electrospinning process can be achieved using a variety of natural and synthetic biomaterials. The electrospinning process technology, along with its utility in the tissue engineering of various tissues, has been systematically reviewed [2,9]. Recent progress and possible future perspectives of electrospun nanofibrous scaffolds for bone tissue engineering, including drug/growth factor delivery for bone tissue regeneration, have been the focus of another recent review paper [10]. Nemati et al. [11] highlighted the latest achievements in the fabrication of electrospun nanofibers, described various ways of modifying the surface and structure of scaffolds to promote their functionality, and also summarized the application of advanced polymeric nanofibrous scaffolds in the regeneration of human bone, cartilage, vascular tissues, and tendons/ligaments. Recently, Zulkifli et al. [12] provided an overview of the electrospinning techniques used to prepare nanofibers for tissue engineering applications. The authors described current research on nanofibers fabrication and characterization, including the main limitations of electrospinning and some possible solutions to overcome these limitations. Udomluck et al. [13] described the details of recently developed fabrication and post-modification techniques and discussed the advanced applications and impact of the integrated system of nanofiber-based scaffolds in the field of bone tissue engineering.

Among the various types of scaffolds, highly porous nanofibrous scaffolds are a potential candidate for supporting cell functions, such as adhesion and proliferation, delivering growth factors, and forming new tissue. Recent reports suggest that the chemical and physical properties of nanofibers can be tuned by post-spinning modification to regenerate specific target tissues. The possibilities of functionalizing electrospun nanofibers for bone tissue engineering to make them more conducive and effective for bone regeneration have also been recently reviewed by Yan et al. [14]. Reactive electrospinning recently gained much attention as it can fabricate fibers with tunable mechanical and functional properties by applying different crosslinking strategies and incorporating various chemical or biochemical reactions into the spinning process [3].

Although the electrospinning method has been developed to produce nanofibrous scaffolds, their isotropic structure, low porosity, and small pore size prevent them from being widely used, especially for anisotropic tissues [15]. However, several studies have been published that aim to overcome this limitation. An example of the use of electrospinning for anisotropic tissue engineering is presented by Liu et al. They developed a multiscale anisotropic scaffold that integrates electrospinning with 3D printing techniques to create a heart-on-a-chip platform [16]. Reid et al. further emphasized the importance of architected fibrous scaffolds in anisotropic tissue engineering. Their research supports the notion that electrospinning can be tailored to produce scaffolds [17]. Fibers have been successfully spatially arranged into honeycomb structures using well-shaped 3D microarchitected metal collectors by other authors [18].

Electrospinning generally produces a nonwoven fibrous network with a typical pore size in the range from tens of nanometers to several micrometers, which is too small for the effective ingrowth of cells from most tissues into the scaffold. Therefore, the electrospinning technique has difficulty in directly producing clinically relevant 3D nanofibrous scaffolds with desired structural properties for most tissue engineering applications [15]. In addition, the technology also suffers from other drawbacks, such as the need for specialized equipment, high complexity, high electrical potential, and electrically conductive targets, as well as the very high purchase price. The widespread commercial use of electrospinning is mainly limited by its low production rate. The inability to produce large quantities of nanofibers has been a major obstacle to the further development and commercialization of electrospinning technologies. Erickson et al. [19] presented a high-throughput centrifugal electrospinning (HTP-CES) system capable of producing large numbers of highly-aligned nanofiber samples with high-yield and tunable diameters. Compared to conventional electrospinning techniques, fibers spun with the HTP-CES not only showed superior alignment but also better diameter uniformity. This system can be easily scaled up for industrial production of highly aligned nanofibers with tunable diameters, potentially meeting the requirements of various engineering and biomedical applications.

Centrifugal spinning is an alternative method for producing nanofibers from various materials at high speed and low cost. In 2008, Weitz et al. [20] reported unexpected findings of nanoscale fibers down to 25 nm in diameter emerging from a polymer solution during a standard spin-coating process. The fiber formation relies on the Raleigh–Taylor instability of the spin-coated liquid film, which arises from a competition between the centrifugal force and the Laplace force induced by the surface curvature. This process offers an attractive alternative to electrospinning for the efficient, simple, and nozzle-free production of nanoscale fibers from a variety of polymer solutions. In centrifugal spinning, the spinning fluid is introduced into a rotating spinning head. When the rotational speed reaches a critical value, the centrifugal force overcomes the surface tension of the spinning fluid to eject a jet of fluid from the nozzle tip or spinning edge of the spinning head. The jet then undergoes a stretching process and is finally deposited on the collector, forming solidified nanofibers. Centrifugal spinning is simple and enables the rapid production of nanofibers for a wide range of applications. The centrifugal spinning process has been reviewed and compared with conventional nanofiber production methods [21].

Zhang et al. [22] introduced the working principle and development of high-speed centrifugal spinning. The jet motion and nanofiber formation process under the action of centrifugal force are explained in detail. The effects of equipment parameters and spinning solution parameters on the final nanofiber morphology are presented. These parameters are controllable and include spinneret rotational speed, nozzle length and diameter, spinning solution concentration, spinning solution viscosity and surface tension, and collection distance. This approach has been applied to various synthetic and natural polymers [19,23,24,25], and even to the creation of ceramic nanofibers [26]. This technique does not use a high-voltage field and is therefore safer than electrospinning. It is also faster and has a higher throughput [27].

A brief history of the centrifugal spinning method has also been recently reviewed [28]. The centrifugal spinning method stands out for its simplicity, its high rate of fiber production, its flexibility, and the ease of its implementation when compared to the other methods. The high potential of the technique for use in the biomedical field has been emphasized. Marjuban et al. [29] provided an overview of the current advances in centrifugally spun polymeric fiber-based materials and their morphological features, performance, and characteristics for tissue engineering applications. Their paper presents the fundamental process of fiber generation and the effects of fabrication parameters (machine, solution) on morphologies such as fiber diameter, distribution, alignment, porous features, and mechanical properties.

A comparative study of conventional electrospinning, centrifugal spinning, and the basics of electrocentrifugal spinning has recently been published [30]. The surface tension and viscosity of the solutions are the spinnability determinants in these three methods, which are affected by the type of polymers and solvents and also by the concentration of the solution and have to be overcome through electrical or centrifugal forces or both. The effective parameters of the process and the fiber morphology are investigated for each of the three methods in the aforementioned paper.

Simultaneous centrifugal spinning and solution blowing to form polymer nanofibers has also been reported [31]. The process is capable of producing 6 kg of fibers per hour and therefore offers mass production capabilities compared to other established polymer nanofiber generation methods such as electrospinning, centrifugal spinning, and blowing.

In industrial practice, electrospinning is mainly used to produce nanofibers, but centrifugal spinning and combined centrifugal spinning/electrospinning technologies are also used, albeit marginally and with different specificities for each method. We consider melt blowing, which can be tuned to produce submicron fibers, to be another technology with great potential for the production of nanofiber mats for tissue engineering as it does not require the use of organic solvents and is quite environmentally friendly. However, melt-blowing technologies are limited to the use of thermoplastic polymers, such as polylactic acid (PLA), polycaprolactone (PCL), polyglycolic acid (PGA), polylactic glycolic acid (PLGA), polyhydroxyalkanoates (PHA), or polybutylene succinate (PBS).

As mentioned above, commercial electrospinning typically produces nanofibrous meshes that are relatively thin and have a two-dimensional (2D) morphology rather than the three-dimensional (3D) structure required for the reconstruction of most tissues. Control of the pore structure is an important aspect of scaffold fabrication as it directly affects cell infiltration. Small average pore sizes result in reduced cellular infiltration. In some cases, infiltration can be limited to a very thin layer of cells on top of the nanofibrous scaffold. This phenomenon limits the potential benefits of nanofibers for certain tissue engineering applications. The importance of the pore structure can be seen when comparing microfibrous and nanofibrous constructs. In some cases, the larger pore size of microfibrous scaffolds has been shown to promote higher levels of stem cell differentiation, in addition to improved cell infiltration [32].

Due to the aforementioned shortcomings of using conventional electrospinning, we have explored an alternative approach, namely, the method of centrifugal spinning. Our preliminary results have shown that centrifugal spinning is capable of producing 3D fibrous constructs. The aim of our study was to verify the possibility of using our own patented nozzleless centrifugal spinning technology [33,34] to fabricate micro/nanofibrous scaffolds for special fat substitutes from several biocompatible and biodegradable polymers. Adipose tissue engineering using nanofibrous scaffolds presents several challenges that must be addressed to improve the efficacy of tissue regeneration. One important area of concern is the mechanical properties of the scaffolds. Many nanofibrous scaffolds, particularly those made from synthetic polymers, tend to exhibit rigidity, which can hinder their application in soft tissue engineering, such as adipose tissue regeneration. The mechanical mismatch between the scaffold and the native adipose tissue may result in inadequate support for cell proliferation and differentiation, ultimately affecting tissue integration and functionality [35]. Therefore, the aim of our study was to prepare soft fibrous scaffolds with a consistency similar to adipose tissue and larger pore sizes than nanofibrous networks prepared via conventional electrospinning.

We have created a bioartificial adipose tissue substitute using 3D micro/nanofibrous scaffolds in combination with human adipose-derived stem cells (ASCs), which colonized these scaffolds and were able to differentiate into adipocytes (see Supplementary Materials, Figures S1–S7). These adipose tissue analogues could be used in the future instead of classical fat grafts, applied, e.g., for breast reconstruction after a cancer ablation procedure. We anticipate that our cell–material constructs could reduce postoperative atrophy.

## 2. Materials and Methods

### 2.1. Preparation of Fibrous Scaffolds

**Figure 1:**
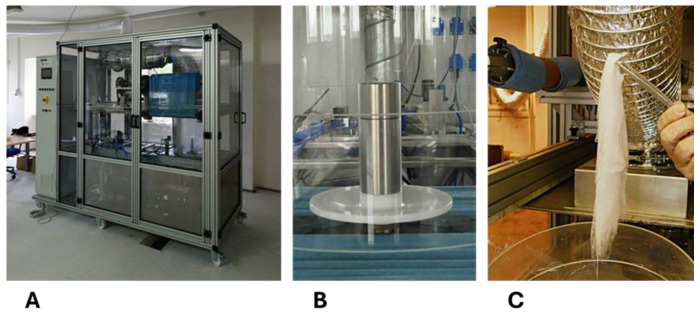
The NANOCENT demonstrator (DBH Technologies, Czech Republic) for the centrifugal spinning technology used to produce the micro/nanofibrous fiber scaffolds (**A**), equipped with the two-piece plastic disc (**B**). Three-dimensional fiber construct (**C**).

The NANOCENT centrifugal spinning demonstrator (DBH Technologies, Černčice, Czech Republic) was used to fabricate the nanofibrous scaffolds (see Figure 1A). The demonstrator is based on a new method for producing nano/microfibers and can be adapted to produce different types of fiber membranes and 3D constructs for use in a wide range of potential applications. The production of micro/nanofibrous scaffolds is based on a industrially protected centrifugal technology [33,34], using a fiber-forming conical hollow disc, composed of a lower and an upper part forming an inner space with an outlet gap in between, connected to a hollow shaft and rotating around the axis at 1000–5000 rpm. A scheme of the equipment is available in the Supplementary File (Figure S10). A polymer solution is sprayed through orifices from the interior of the hollow shaft into the interior of the rotating disc. The fibers are formed by centrifugal force at the edge of the outlet gap and can be collected in the form of homogeneous mats on a cylindrical collector positioned next to the fiber-forming disc or using other types of collectors. The cotton wool-like fiber constructs (referred to as the 3D fiber constructs in the following text) used as scaffolds in our study were collected on the inner walls of the polyacrylate working tube of the Nanocent demonstrator (see Figure 1B,C). A two-piece plastic disc was used for all the experiments (see Figure 1B).

Poly(3-hydroxybutyrate) (PHB) (Biomer, Krailling, Germany), polylactide (PLA) Ingeo™ 4043D (NatureWorks, Plymouth, MA, USA), poly(1,4-butylene succinate) (PBS), extended with 1,6-diisocyanatohexane (Sigma Aldrich, Prague, Czech Republic) and polycaprolactone (PCL), average Mn 80,000 (Sigma Aldrich, Prague, Czech Republic) were used in the experiments. All the polymers and their mixtures in chloroform (CF) were dissolved at 70 °C under continuous shaking for 48 h. The concentrations of individual polymers and their mixtures in CF and the parameters of the fiber formation process are summarized in Table 1.

### 2.2. Characterization of the Scaffold Microstructures by Scanning Electron Microscopy

Scanning electron microscopy (SEM) was used to analyze the particle size and morphology:

(1) Fiber samples were fixed to the holders with double-sided adhesive tape and were examined with a Phenom G2 scanning electron microscope (Phenom-World BV, Eindhoven, The Netherlands).
(2) SEM scans were acquired via a scanning electron microscope (FIB-SEM, LYRA3 GMU, Tescan, Brno, Czech Republic). The applied acceleration voltage was 10 kV. The investigated samples were covered with a Pt conductive layer.

### 2.3. Brunauer–Emmett–Teller (BET) Analysis

Specific surface area and pore volume were determined from the adsorption and desorption isotherms on a NOVA3200 (Quantachrome Instruments, Boynton Beach, FL, USA) using Quantachrome NovaWin software. Samples were degassed for 24 h at 100 °C, and then nitrogen adsorption and desorption isotherms were recorded (Linde, Guildford, UK, 99.999%). Five-point BET analysis was used to determine specific surface area, and the 40-point Barrett–Joyner–Halenda (BJH) was used to determine pore volume. Each sample was measured four times with an experimental error of 5%.

### 2.4. Sterilization of the Prepared Fibrous Scaffolds

Before the experiments, the prepared scaffolds were sterilized by gamma-irradiation using the basic sterilization dose (Microtron MT25, Nuclear Physics Institute, Academy of Sciences of the Czech Republic, Řež near Prague).

### 2.5. Cellular Component, Isolation, and Characterization

The biocompatibility and permeability for the cell colonization of the prepared samples were tested with adult human mesenchymal stem cells. Adipose-derived stem cells (ASCs) were chosen because they can be differentiated into many cell types of mesenchymal origin, including chondrocytes, osteoblasts, smooth muscle cells, and especially adipocytes, of which ASCs are de facto precursors (see Supplementary Materials, Figures S1–S7). ASCs were isolated from lipoaspirates of subcutaneous abdominal adipose tissue of a healthy female donor (age 40 years) in accordance with the tenets of the Declaration of Helsinki on experiments involving human tissues, under the approval of the Ethics Committee of the Na Bulovce Hospital, Prague, Czech Republic, and with written informed consent obtained from the donor prior to liposuction.

The isolation procedure was based on the original procedure published by Estes et al. [36] and further modified in our previous studies [37,38]. The ASCs were then used in passages 2–3, previously characterized by flow cytometry (Accuri C6 flow cytometer) to confirm the presence of typical mesenchymal stem cell markers (CD105, CD90, CD73, CD29) and the absence of markers of other cell types, such as hematopoietic cells and endothelial cells (CD45, CD34, CD31). The percentage of stem cell-specific CD markers was mostly higher than 98%. A more detailed description of the isolation, cultivation, and characterization of ASCs can be found in our previous studies [37,38].

### 2.6. Cell Cultivation

The sterile scaffolds were cut into 10 × 10 × 3 mm samples, soaked in the culture medium (see below), placed in 24-well polystyrene plates (Techno Plastic Products, Trasadingen, Switzerland, inner well diameter 1.5 cm), and seeded with ASCs at a density of 300,000 cells/sample. The cells on the samples were cultured for 10 days in a Dulbecco’s modified Eagle’s medium (DMEM; Sigma-Aldrich, St. Louis, MO, USA) and supplemented with 10% fetal bovine serum (FBS; Sebak GmbH, Aidenbach, Germany) and gentamicin (40 μg/mL, LEK, Ljubljana, Slovenia) at 37 °C in a humidified air atmosphere containing 5% CO_2_. Three-to-four independent samples were tested for each scaffold type. The cell behavior on the samples was evaluated on days 2, 6, and 10 after seeding.

### 2.7. Cell Visualization and Confocal Microscopy

Cell morphology, cell spreading on the scaffold fibers, scaffold overgrowth by cells, and cell ingrowth within the scaffolds were evaluated in cells visualized by fluorescence staining of the filamentous actin (F-actin). Prior to staining, the samples were fixed with 4% paraformaldehyde in phosphate-buffered saline for 30 min at room temperature. The samples were permeabilized with 0.1% Triton X-100 and 1% bovine serum albumin in phosphate-buffered saline for 20 min, followed by incubation in 1% Tween-20 in phosphate-buffered saline for 20 min. F-actin was then stained with Alexa Fluor 568-conjugated phalloidin (Thermo Fisher Scientific, Cat. No. A12380, 1000× diluted) for 1 h at room temperature. Cell nuclei were counterstained with 4’,6-diamidin-2-fenylindol (DAPI; Sigma-Aldrich, 32670, 20 μg/mL), added to the phalloidin solution. Microimages were captured using an Andor Dragonfly 503 scanning disc confocal microscope equipped with a Zyla 4.2 PLUS sCMOS camera (Andor Technology Ltd., Belfast, UK) and an HC PL APO 20x/ 0.75 IMM CORR CS2 objective, allowing for 3D visualization of the constructs and measurement of the depth of cell penetration into the scaffolds.

## 3. Results

### 3.1. Fibrous Scaffolds

In our study, the 3D fiber constructs were prepared from the single polymers (PLA, PHB, PBS, and PCL) or several blends of the polymers (PLA/PHB 4/1; PHB/PBS 1/4, 2/7; PLA/PCL 5/1, 13.5/4 (*w*/*w*)). The centrifugal spinning parameters of the individual polymers and the polymer blends using the Nanocent Demonstrator are summarized in Table 1. Figure 1 shows the NANOCENT Demonstrator (DBH Technologies, Czech Republic) for the nozzleless centrifugal spinning technology used to produce the micro/nanofibrous scaffolds (Figure 1A), equipped with the two-piece plastic disc (Figure 1B). The 3D fiber constructs prepared by the technology described above is shown in Figure 1C.

### 3.2. Characterization of the Scaffold Microstructure by SEM

#### 3.2.1. Microstructure of the 3D Fiber Constructs from the Individual Polymers

**Figure 2:**
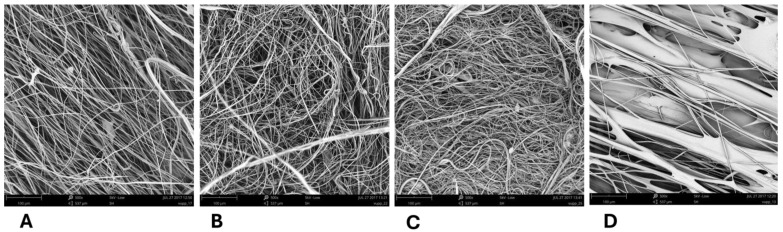
SEM images of the 3D fiber constructs prepared from the single polymers. Scale bars: 100 µm. SEM microscope: Phenom G2, Zeiss, Oberkochen, Germany.

PLA, PHB, and PBS formed a relatively dense network of micro/nanofibers, unsuitable for cell colonization due to their small pore size, typically in the order of units of micrometers, and only exceptionally up to tens of micrometers (see Figure 2). The prepared samples contained a mixture of fibers with diameters in the micrometer and submicrometer range. The PCL itself created a relatively dense network of coarser fibers that also contained a large number of non-fibrous planar structures.

#### 3.2.2. Microstructure of 3D Fiber Constructs from the Polymer Blends

**Figure 3:**
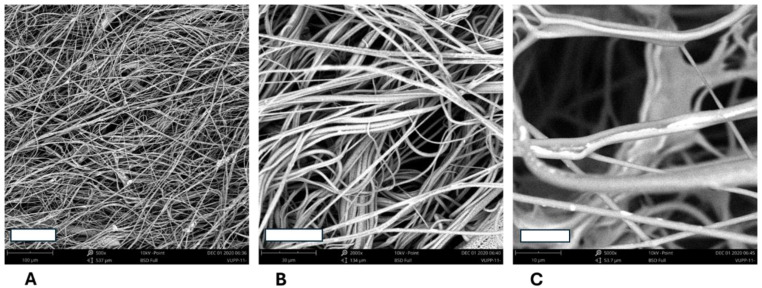
SEM images of the 3D fiber constructs prepared from PLA/PHB blend. Weight ratio: 4/1. Scale bars: 100 µm (**A**), 50 µm (**B**), and 10 µm (**C**). SEM microscope: Phenom G2.

**Figure 4:**
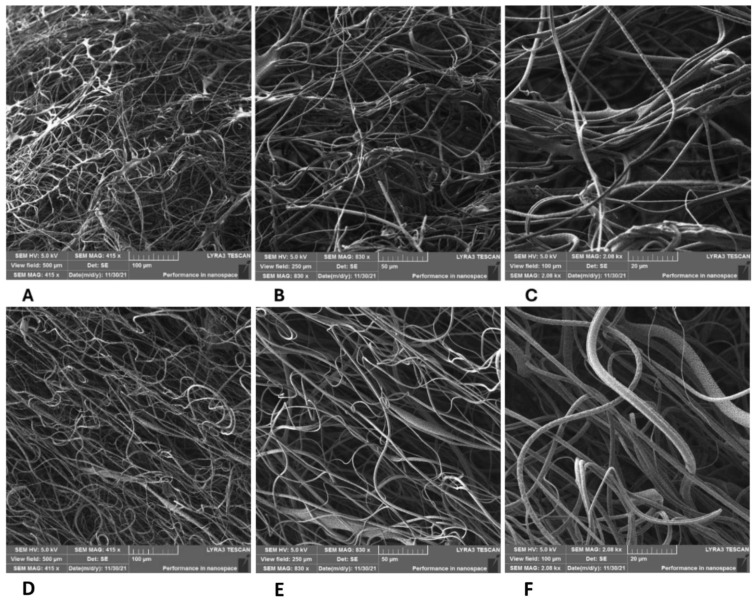
SEM images of the 3D fiber constructs prepared from PHB/PBS blend. Weight ratio: 1/4 (**A**–**C**) or 2/7 (**D**–**F**). Scale bars: 100 µm (**A**,**D**), 50 µm (**B**,**E**), and 20 µm (**C**,**F**). SEM microscope: FIB-SEM, LYRA3 GMU, Tescan.

**Figure 5:**
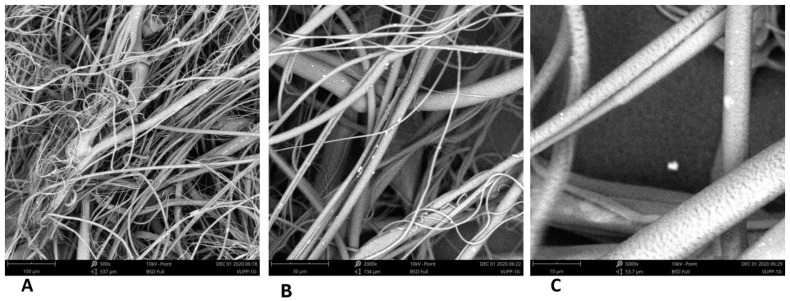
SEM images of the 3D fiber constructs prepared from PLA/PCL blend. Weight ratio: 5/1; disk rotation speed: 1500 rpm. Scale bars: 100 µm (**A**), 30 µm (**B**), and 10 µm (**C**). SEM microscope: Phenom G2.

**Figure 6:**
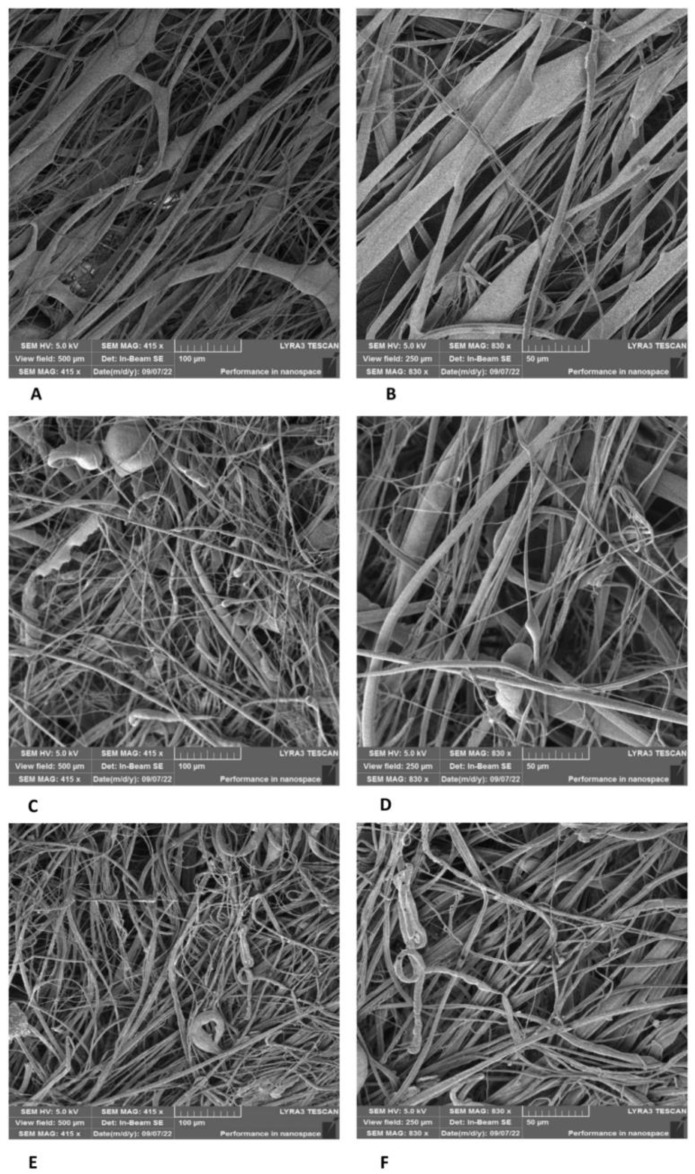
SEM image of the 3D fiber constructs prepared from PLA/PCL blend. Weight ratio: 13.5/4; disk rotation speed: 1500 rpm (**A**,**B**), 3000 rpm (**C**,**D**), or 5000 rpm (**E**,**F**). Scale bars: 100 µm (**A**,**C**,**E**) and B 50 µm (**B**,**D**,**F**). SEM microscope: FIB-SEM, LYRA3 GMU, Tescan.

**Figure 7:**
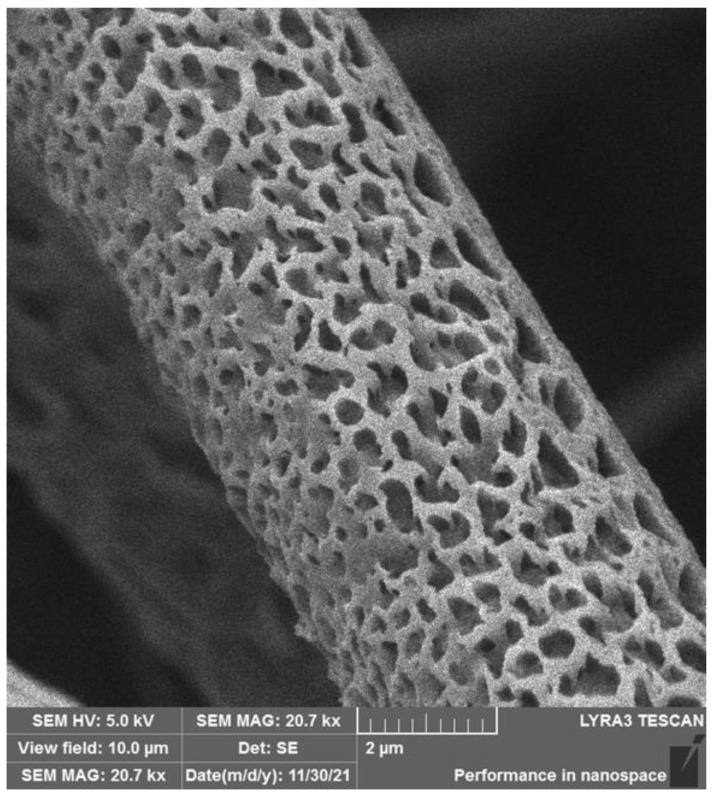
SEM image of a fiber of the 3D construct prepared from PLA/PCL blend. Weight ratio: 13.5/4—A fiber detail. SEM microscope: FIB-SEM, LYRA3 GMU, Tescan.

PLA/PHB, PHB/PCL, and PHB/PBS blends form relatively dense networks of micro/nanofibers with pore sizes again in the order of units to tens of micrometers, regardless of the ratio of each polymer in the blends (see Figure 3 and Figure 4). Larger pores were obtained with the PLA/PCL blend (see Figure 5, Figure 6 and Figure 7), regardless of the tested ratios of both polymers (5/1; 13.5/4 *w*/*w*). We have also tested the influence of the rotation speed of the spinning disc on the pore size of the PLA/PCL 13.5/4 *w*/*w* fibrous networks (see Figure 6A–F). The best results were obtained at a medium rotation speed of 3000 rpm (see Figure 6C,D). Further increases in disc speed have already resulted in a denser fiber structure and a reduction in pore size (see Figure 6E,F). The samples of the 3D fiber constructs prepared from PLA/PCL blends contained a greater amount of microfibers with diameters of several micrometers in combination with submicron fibers with relatively large pore sizes of up to tens of micrometers. We observed no evidence of degradation of the polymer fibers by gamma irradiation in the SEM images (see Figure 6).

Figure 7 shows a detail of the 13.5/4 (*w*/*w*) PLA/PCL microfiber, where the internal microporosity of the individual fibers is clearly visible. The pore size visible in Figure 7 is in the order of hundreds of nanometers. The potential influence of the internal microporosity of the individual fibers on the water retention of the fibrous scaffolds and cell attachment to the scaffold fibers is discussed below.

Table 2 shows the results of the determination of the influence of the rotational speed on the fiber diameter of PLA/PCL 13.5/4. We found no significant effect of the rotational speed of the spinning disk (rpm) on the median fiber diameter (µm) (see Table 2). The median fiber diameter ranged from 5.71 to 7.81 µm. All samples of the 3D fiber constructs contained a mixture of submicron fibers and microfibers with a very wide distribution of fiber diameter values. A figure (Figure S11) with histograms of the fiber diameter distributions at different rotational speeds of the spinning disk is available in the Supplementary File.

### 3.3. BET Analysis

#### BET Analysis of PLA/PCL 3D Fiber Constructs

**Figure 8:**
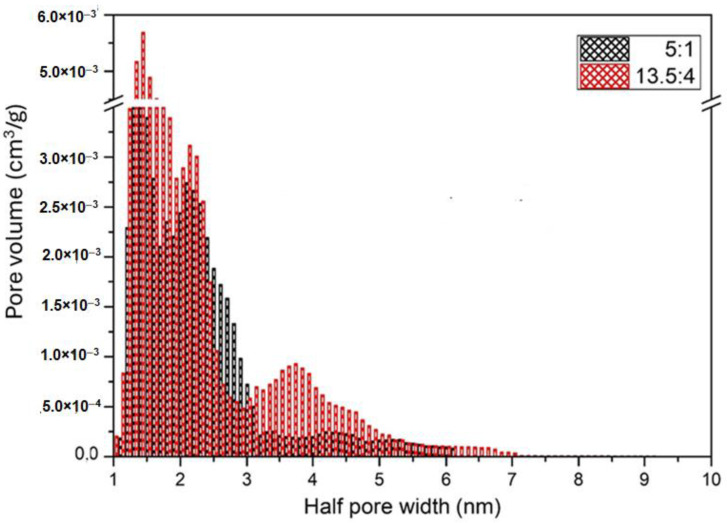
Results of BET analysis of the micro/nanofibrous scaffolds prepared from the 5/1 and 13.5/4 (*w*/*w*) PLA/PCL blends: pore size distribution histograms.

**Figure 9:**
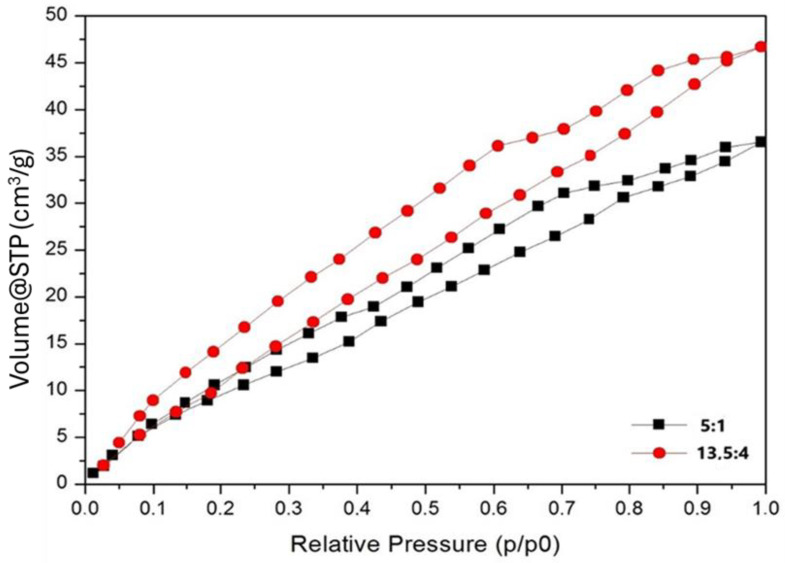
Results of BET analysis of the micro/nanofibrous scaffolds prepared from the 5/1 and 13.5/4 (*w*/*w*) PLA/PCL blends: nitrogen adsorption and desorption isotherms.

The BET analysis results of our micro/nanofibrous biodegradable scaffolds are shown in Table 3 and Figure 8 and Figure 9. The specific surface areas of the PLA and PHB fibrous constructs are relatively low due to the high content of microfibers and relatively high pore sizes in the fibrous networks, i.e., in the range of approximately 12 to 16 m^2^·g^−1^. The BJH pore sizes, corresponding to the nanoporosity of the pore size of 2–50 nm, are in the range of 0.013–0.018 cm^3^·g^−1^. However, the addition of PLA to PCL resulted in a significant increase in both the specific surface area and BJH pore size, which was particularly evident in the PLA/PCL 13.5/4 scaffolds in comparison with PLA/PCL 5/1 scaffolds (Figure 8 and Figure 9). Therefore, in addition to the microporosity of the individual fibers visible in the SEM images (pore size in the order of hundreds of nanometers; see Figure 7), the BET analysis revealed the internal nanoporosity of the fibers in the order of units of nanometers. The BET analysis of material porosity is limited to the range of pore sizes from about 2 nm to 20 nm and is unable to detect smaller and larger pores. Therefore, only the combined results of SEM and BET analyses revealed the hierarchical porosity of the polymeric micro/nanofibers produced via the centrifugal spinning. The hierarchical porosity of the produced polymeric micro/nanofibrous scaffolds can significantly influence their water retention ability and fiber roughness, as discussed in more detail below.

### 3.4. Biocompatibility and Cell Colonization of the Prepared Scaffolds

#### 3.4.1. Comparison of Cell Colonization of Selected Scaffolds

We investigated the biocompatibility of nanofibrous scaffolds prepared from single polymers (PLA, PHB) and different blends of these polymers with PBS or PCL (PHB/PBS 1/4, 2/7; PLA/PHB 4/1; PLA/PCL 5/1; 13.,5/4 (*w*/*w*)). We tested the colonization of five different scaffolds with ASCs during 10 days of cultivation time. We visualized the cells via fluorescence staining and monitored the adhesion, growth, and survival of the cells on the tested samples, as well as the penetration of the cells into the scaffolds. The comparison of cell ingrowth thickness in different scaffold types is shown in Table 4.

**Figure 10:**
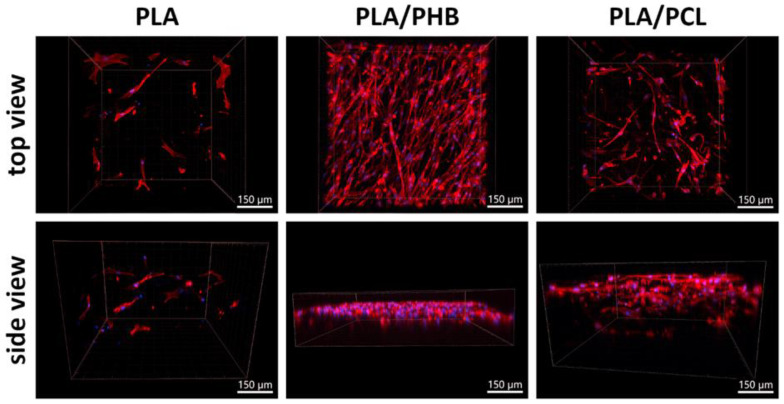
ASC colonization of nanofibrous scaffolds composed of PLA, PLA/PHB 4/1 *w*/*w*, and PLA/PCL 13.5/4 (*w*/*w*) on day 10 after seeding. Fluorescence staining of F-actin in cells with phalloidin (red), and cell nuclei counterstained with DAPI (blue). Images were taken either from the top of the scaffolds or as a 3D side view. Andor Dragonfly 503 confocal microscope, obj. 20×, scale bars 150 µm.

The single polymer scaffolds were usually colonized by cells only on the surface, and even with prolonged cultivation, there was no penetration of cells to a greater depth (the maximum cell penetration depth was 427 ± 98 µm; Table 4). From this point of view, polymer blends are preferable. Cell visualization to illustrate the depth of cell penetration into three selected scaffolds, i.e., a single polymer scaffold (PLA), and two blends, i.e., PLA/PHB 4/1 *w*/*w* and PLA/PCL 13.5/4 *w*/*w*, was performed via fluorescent staining with phalloidin. Cell adhesion, spreading, proliferation, and scaffold colonization differed between the different materials. The situation on day 10 is shown in Figure 10. The PLA scaffolds were very poorly colonized, with only a few cells visible in the top view of the scaffolds. The side view showed that cells had penetrated into the material but at a very low density. The PLA/PHB scaffolds were the most densely populated, but the cells remained mostly on the surface of the scaffolds where they formed a dense crust with almost no cell penetration inside the scaffolds. In contrast, the PLA/PCL scaffolds were less colonized with cells on their surface, but a significant number of cells migrated into the interior of the scaffolds and colonized them in depth. Cells penetrated the deepest into the PLA/PCL 13.5/4 *w*/*w* scaffold, as shown in Table 4. Therefore, this scaffold was selected for further investigation.

#### 3.4.2. Time Course of PLA/PCL Scaffold Colonization

**Figure 11:**
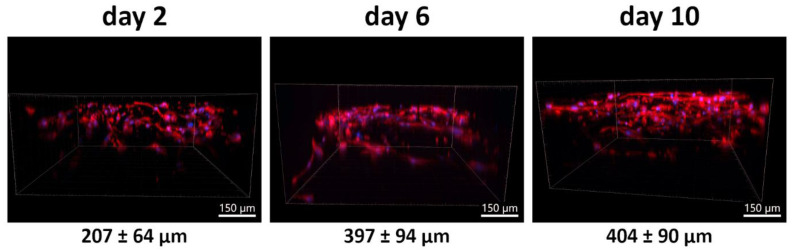
The penetration depth of ASCs in PLA/PCL 13.5/4 (*w*/*w*) micro/nanofibrous scaffolds on days 2, 6, and 10 after cell seeding. Fluorescence staining of F-actin in cells with phalloidin (red), and nuclei counterstained with DAPI (blue). Images were taken as 3D side views. Andor Dragonfly 503 confocal microscope, obj. 20×, scale bar 150 µm.

**Figure 12:**
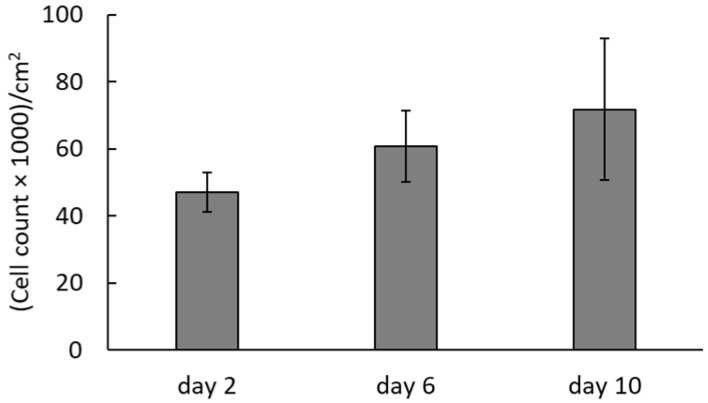
The population density of ASCs on the surface PLA/PCL 13.5/4 (*w*/*w*) scaffolds on days 2, 6, and 10 after seeding. Mean ± S.D. from four images. ANOVA, Student–Newman–Keuls method. No significant differences in cell number were detected.

**Figure 13:**
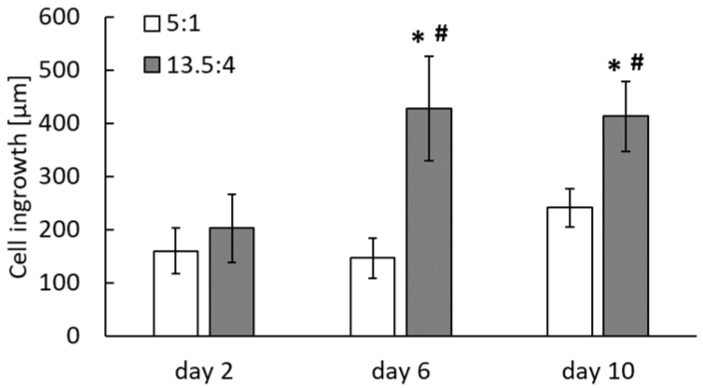
The time course of ASC penetration into PLA/PCL 13.5/4 (*w*/*w*) and PLA/PCL 5/1 (*w*/*w*) scaffolds on days 2, 6, and 10 after seeding. Mean ± S.D. from two–three images for each experimental group and time interval. ANOVA, Student–Newman–Keuls method. *: *p* ≤ 0.05 compared to day 2; #: *p* ≤ 0.05 compared to PLA/PCL 5/1 (*w*/*w*).

Since the PLA/PCL 13.5/4 (*w*/*w*) scaffolds proved to be the most suitable for colonization with ASCs due to the deepest cell penetration, we tested their colonization with ACS over the course of 10 days. Samples were evaluated on day 2, day 6, and day 10 after seeding (Figure 11). Surprisingly, when the population density on the scaffold surfaces was counted on maximum projection confocal microscopy images, it did not change significantly from day 2 to day 10, although it increased slightly on average (Figure 12). However, we observed a marked progression in the depth of ASC colonization. After 10 days, the cells were visible in the cell–material construct to a depth of nearly 500 µm, which is the maximum depth that can be resolved with a given microscope (see Figure 11 and Figure 13).

Quantitative measurements of cell penetration depth from confocal microscopy images showed that cells penetrated statistically significantly deeper on day 6 after seeding than on day 2 after seeding, although this depth no longer changed statistically significantly on day 10 (Figure 13). However, at both day 6 and day 10 after seeding, the depth of cell penetration was greater for the scaffold with a PLA/PCL ratio of 13.5/4 *w*/*w* than for the scaffold with a different PLA/PCL ratio of 5/1 *w*/*w* (Figure 13).

**Figure 14:**
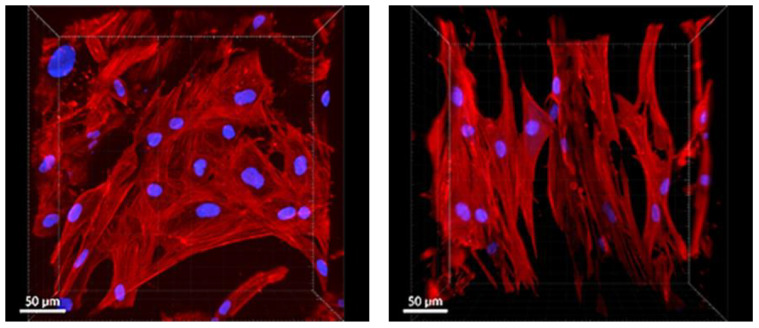
Morphology of ASCs on the PLA/PCL 13.5/4 (*w*/*w*) micro/nanofibrous scaffolds on day 6 after cell seeding. Actin filaments–phalloidin (red); cell nuclei–DAPI (blue). Andor Dragonfly 503 confocal microscope, obj. 20×, zoom 2×, scale bars 50 µm.

Cells on PLA/PCL 13.5/4 (*w*/*w*) scaffolds were well-spread with either a polygonal or elongated morphology typical of well-adhered cells at partial confluence (Figure 14). Cells were often elongated along the fibers of the scaffolds.

#### 3.4.3. Suitability of the Scaffolds from a Biological Point of View

The predicted suitability of the scaffold for use in tissue engineering depends on whether the scaffold is colonized by the appropriate cell type. This colonization depends on the pore size, which can be determined via SEM. The actual depth of cell penetration into the scaffold can be continuously monitored and determined via confocal microscopy, as we have used in our previous work [39]. Centrifugal spinning of single polymers, such as PLA, PHB, or PCL, formed relatively dense networks of micro/nanofibers with pore sizes in the order of units to tens of micrometers, which were less suitable for cell colonization. PLA/PHB, PHB/PCL, and PHB/PBS blends again formed similarly dense networks, regardless of the ratio of individual polymers in the blends (see Figure 3 and Figure 4). Larger pores were obtained with the PLA/PCL blends (see Figure 5, Figure 6 and Figure 7), regardless of the tested ratios of both polymers (5/1; 13.5/4 *w*/*w*). The scaffold permeability to cells, i.e., the comparison of the depth to which the cells migrated inside the scaffolds, confirmed the highest suitability of the PLA/PCL 13.5/4 (*w*/*w*) fibrous scaffold, which showed the deepest ingrowth of ASCs. This result is consistent with the assumption that 3D scaffolds with a high porosity, a large pore size, a large specific surface area, and nanoporosity of the fibers are most suitable for use as a component of adipose tissue substitutes that could reduce postoperative tissue atrophy after reconstructive surgery. Adipose tissue constructs produced in this way could be used in the future instead of conventional fat grafts, for example, in breast reconstruction after cancer ablation.

**Figure 15:**
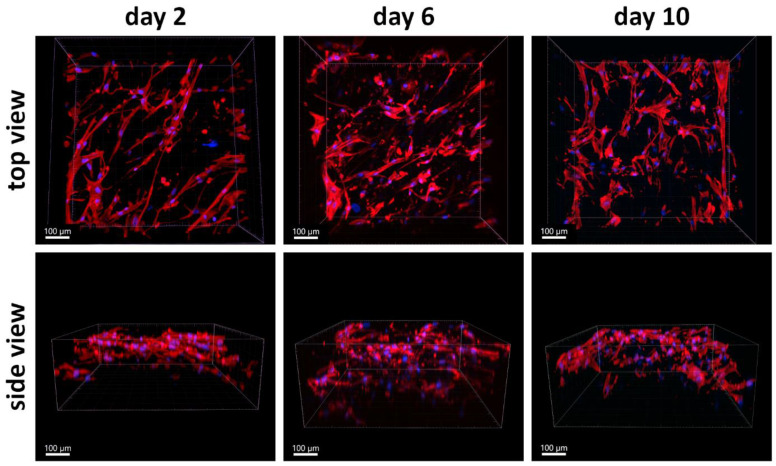
Colonization of the PLA/PCL 13.5/4 (*w*/*w*) micro/nanofibrous scaffolds with ASCs on days 2, 6, and 10 of cultivation in an adipogenic medium. Fluorescence staining of F-actin in cells with phalloidin (red), and nuclei counterstained with DAPI (blue). Images were taken either from the top of the scaffolds or as a 3D side view. Andor Dragonfly 503 confocal microscope, obj. 20×, scale bars 100 µm.

Therefore, we also tested the growth of ASCs on scaffolds in adipogenic medium containing DMEM supplemented with 10% of FBS, 1 μM dexamethasone, 20 μM indomethacin, 10 μg/mL insulin, and 500 μM 3-isobutyl-1-methylxanthine (IBMX). We obtained similar results as in the standard culture medium; i.e., the cells were able to grow on the surface of the scaffolds, as well as to penetrate into their depths (see Figure 15).

## 4. Discussion

### 4.1. Use of PLA and PCL Composite Fibers and Optimal PLA/PCL Ratio

The use of 3D constructs prepared from PLA and PCL composite nanofibers and/or microfibers as biodegradable cell scaffolds for tissue engineering has already been described in a number of publications. PLA and PCL have been shown to be ideal for nanofiber fabrication in various biomedical applications due to their favorable properties, such as their biodegradability and their ability to promote cell growth, similar to native tissues. The optimal ratio of PLA and PCL in tissue engineering scaffolds is the subject of considerable research as it significantly influences the mechanical properties, degradation rates, and biocompatibility of the resulting materials. Several studies have investigated different PLA/PCL ratios to determine the most effective combinations for specific tissue engineering applications. Research indicates that a 1:1 weight ratio of PLA to PCL is often optimal for achieving a balance between mechanical strength and flexibility. For instance, Liao et al. [40] found that electrospun PLA/PCL blend fibers prepared at a 1/1 ratio exhibited defect-free morphology, uniform diameter, and high ductility, making them suitable for tissue engineering applications. This ratio allows the beneficial properties of both polymers to be utilized, with PLA contributing to mechanical strength and PCL improving flexibility and degradation rates. In addition, Xu et al. [41] reported that the incorporation of PCL into PLA scaffolds improved the overall mechanical properties and biocompatibility, suggesting that a blend ratio of approximately PLA/PCL 7/3 may also be effective for certain applications. However, the specific findings regarding the optimal ratio for mechanical properties were not directly addressed in their study, which focused primarily on PCL scaffolds for bone tissue engineering. Moreover, Hairaldin et al. [42] found that a 9/1 ratio of PLA to PCL had superior mechanical properties compared to other ratios, highlighting the importance of optimizing the blend for specific mechanical requirements. This suggests that while a 1/1 ratio is often cited as optimal for general applications, specific tissue types may benefit from different ratios based on the mechanical and degradation properties required. Pisani et al. [43] prepared electrospun matrices suitable for circular replacement of esophageal defects from poly-L-lactide-co-poly-ε-caprolactone 7/3 ratio and characterized their physicochemical and functional properties. PLA and PCL composite nanofibers have also been used to construct biodegradable artificial Anterior Cruciate Ligament (ACL) scaffolds, which must withstand mechanical stress during neoligament formation and stabilize the ACL [44]. The purpose of their study was to determine the effect of compositional variations in PLA and PCL on ultimate tensile strength and modulus of elasticity, fiber diameter, cytotoxicity level, degradation time, and the tissue cell viability. The ideal scaffold was composed of PLA-PCL 8/2 (*w*/*w*), with an average fiber diameter of 827 ± 271 nm and a degradation time of approximately 219 days in phosphate-buffered saline solution. The higher the PCL, the lower the percentage of viable cells and the faster the degradation of the specimen.

However, in our study, the mechanical properties of our newly developed scaffolds could not be tested because a defined specimen size is required to determine mechanical properties, which was not feasible due to their high malleability and deformability. Our scaffold specimens were designed to be suitable for specific fat substitutes, i.e., very ductile, soft, and flexible. At the same time, our scaffolds are not a homogeneous nanofibrous material (see Figure 6 and Figure S11) because, for a soft tissue construct, it is appropriate for some of the fibers to be thicker and stiffer and to serve more as a support fiber for the entire construct. Cells can settle and migrate on the submicron fibers of varying thickness. Large interfiber spaces allow for the migration of cells and eventual ingrowth of nourishing vessels. Nevertheless, these material properties make objective measurement of mechanical properties very difficult, if not impossible. However, we did not observe any changes in mechanical properties during the experiments by subjective evaluation.

To develop more clinically relevant 3D scaffolds, electrospun nanofibrous scaffolds with different weight ratios of PCL/PLA were fabricated via the thermally induced (nanofiber) self-agglomeration (TISA) method [45]. The results indicated that all of the 3D scaffolds were elastic/resilient and possessed interconnected and hierarchical pores with sizes ranging from sub-micrometers to ~300 μm; therefore, the morphological structures of these scaffolds were similar to those of natural ECMs. The scaffolds with PLA/PCL 4/1 (*w*/*w*) ratio exhibited the best overall properties in terms of density, porosity, water absorption capacity, mechanical properties, bioactivity, and cell viability. Furthermore, alkaline phosphatase (ALP) activity, calcium content, and gene expression levels also increased with the increasing PLA weight ratio, likely due to the improved stiffness/bioactivity of the scaffold. By increasing the weight ratio of stiffer and more bioactive PLA in the 3D PCL/PLA blend scaffolds, the osteogenic differentiation of human mesenchymal stem cells (hMSCs) was enhanced. Future studies should continue to explore PLA/PCL ratios in conjunction with specific cell types and tissue engineering goals to further refine the optimal formulations. Both PCL and PLA nanofibrous scaffolds were safe and non-toxic to the cells evaluated, and both scaffolds supported cell attachment and proliferation of bone marrow and adipose tissue-derived mesenchymal stem cells [46].

The fabrication of a novel three-dimensional porous PCL/PLA tissue engineering scaffold with high connectivity for endothelial cell migration was also described. PLA electrospun membranes and PCL/PLA (70/30) films were first fused via hot pressing to obtain a composite film containing nanofibers. Supercritical fluid microfoaming technology was used to fabricate a nanofiber structure and a highly interconnected porous scaffold. Cross-sectional human umbilical endothelial cell (HUVEC) culture results showed that the scaffolds were biocompatible and significantly promoted the adhesion, migration, and penetration of HUVECs. A single-cell migration assays showed that the 3D porous scaffolds effectively promoted HUVEC migration [47]. Therefore, 3D porous PCL/PLA scaffolds have shown significant potential in the field of vascular patches. Dong et al. [15] used a modified electrospinning receiving system with a rotating mandrel and a water bath to fabricate a micro/nanofibrous PLA/PCL scaffold with anisotropic structure. The modified system promotes porosity and increased pore size. Compared with the nanofibrous scaffold prepared by the conventional electrospinning system, the scaffold consisted of oriented microfibers and random nanofibers. PLA/PCL electrospun membranes with tailored fiber diameters have also been used as drug delivery systems [48]. Poly(caprolactone-co-polylactic acid) nanofibrous scaffold in combination with 5-azacytidine and transforming growth factor-β to induce cardiomyocyte differentiation of adipose-derived mesenchymal stem cells was described by Tambrchi et al. [49].

While a 1/1 ratio of PLA to PCL is often reported as optimal for many tissue engineering applications due to its balanced properties, variations such as 7/3 or 9/1 may also be advantageous depending on the specific requirements of the tissue being engineered.

### 4.2. Influence of Fiber and Pore Size on Cell Colonization

Although it is well known that centrifugal spinning can produce pores in nanofibrous structures that are up to an order of magnitude larger than those produced by the commonly used electrospinning method [50], the pore size is often still too small for effective colonization of the interior of nanofibrous structures. The optimal pore size in scaffold fiber networks has been extensively discussed in the scientific literature. A study by Shanmugasundaram et al. [32] evaluated the effect of scaffold design, specifically examining the effects of a range of nano- to micrometer-sized fibers, as well as the resulting pore size and mechanical properties, on the chondrogenic differentiation of human mesenchymal stem cells (MSCs) derived from the adult bone marrow. Chondrogenic markers, i.e., aggrecan, chondroadherin, Sox9 transcription factor, and collagen type II, were highest for cells on micrometer-sized fibers (5 and 9 μm) with pore sizes of 27 and 29 μm, respectively, compared to cells on nano-sized fibers (300 nm and 600 to 1400 nm) with pore sizes of 2 and 3 μm, respectively. Micron-sized fibers with large pore structures and mechanical properties comparable to the cartilage ECM enhanced chondrogenesis.

Pore size in nanofibrous 3D scaffolds is a critical determinant of cell behavior, affecting cell migration, nutrient transport, and overall scaffold functionality in tissue engineering applications. The relationship between pore size and cell behavior is complex, and optimal pore size is essential for effective tissue regeneration. Research indicates that larger pore sizes generally facilitate cell infiltration and proliferation. For example, Guneta et al. [51] demonstrated that scaffolds with pore sizes greater than 100 μm significantly enhanced ASC proliferation, as these dimensions allow for adequate cell migration and nutrient flow. Similarly, Rad et al. [52] reported that their 3D scaffolds, with an average pore sizes greater than 20 μm, provided a suitable environment for cell penetration and proliferation compared to 2D scaffolds, emphasizing the importance of pore size in promoting cellular activities. In addition, Xu et al. [41] highlighted that their electrospun PCL scaffolds had interconnected pores with sizes up to approximately 300 μm, which not only supported cell proliferation but also mimicked the natural ECM. This interconnected pore structure is critical for maintaining a conducive environment for cell growth by allowing the diffusion of nutrients and the removal of metabolic waste, which is essential for maintaining cell viability.

Conversely, pores that are too large may reduce cell adhesion and proliferation. For example, Kim et al. [53] found that while microfibrous scaffolds can create a three-dimensional structure, their excessively large pore sizes can reduce the specific surface area available for cell attachment, thereby inhibiting cell proliferation. This suggests that there is an optimal range of pore sizes that must be maintained to balance the benefits of cell infiltration with the need for adequate surface area for cell adhesion. Furthermore, the specific pore size requirements may vary depending on the cell type involved. For instance, Hsia et al. [54] found that fibroblasts preferred pore sizes of 6–20 μm for optimal growth, while other cell types required larger pores. This variability underscores the importance of tailoring pore sizes to the specific cellular context of the tissue being engineered.

The typical pore size of electrospun nanofiber mats can vary significantly depending on several factors, including the polymer used, the electrospinning parameters, and the specific application for which the nanofiber mat is intended. In general, the pore sizes in electrospun nanofiber mats range from several hundred nanometers to several micrometers. For example, Samie et al. [55] reported that the average pore diameter in their electrospun nanofiber mats was approximately 863 ± 142 nm and 925 ± 124 nm for different samples, indicating a relatively small pore size suitable for applications such as tissue engineering. Similarly, Lembach et al. [56] found that the size of the interfiber pores in electrospun nanofiber mats is typically on the order of a few micrometers. However, Wulkersdorfer et al. [57] showed that by varying the humidity during the electrospinning process, they achieved pore sizes ranging from 900 to 5000 μm^2^, demonstrating the tunability of pore dimensions based on environmental conditions. This flexibility is further supported by Jeong et al. [58], who noted that large pores, typically over a few micrometers, can be obtained using specific electrospinning techniques, although such large pores may limit the applicability of the material in certain contexts.

The use of various additives and techniques can further influence the pore size. A number of strategies have been developed to increase the pore size of electrospun 3D nanofibrous constructs while maintaining their nanoscale features [6]. These include salt leaching techniques, where salt crystals are mixed with the fibers during fabrication and leached out after spinning [59]; formation of ice crystals on the collecting plate, which results in larger pores in the construct after melting the ice crystals [60]; using a dual electrospinning setup, where the additional polymer stream is used to create sacrificial fibers that are eluted after spinning, increasing the void space in the construct [61]; using a spherical collecting dish with metallic pegs dispersed throughout to form an uncompressed, cotton ball-like mesh of nanofibers [62]; or dispersing nanofibers within a microfiber framework [63]. Mahjour et al. [64] discussed methods such as sacrificial microbeads and cryogenic electrospinning to increase porosity and pore size in electrospun matrices, which can be crucial for applications requiring improved cell infiltration. This is echoed by Lee et al. [65], who emphasized that the high porosity and surface area of electrospun nanofibers are advantageous for tissue engineering applications. However, the options for increasing the pore size in electrospun matrices to meet the requirements for successful cell penetration into the materials are limited, and the methods described above are quite complicated, laborious, difficult to reproduce, reduce the mechanical strength of the nanofiber constructs, and often require non-standard design modifications to existing devices.

In summary, the pore size of nanofibrous scaffolds significantly influences cell colonization by affecting cell adhesion, migration, proliferation and nutrient transport. While larger pores generally promote cell infiltration and growth, pores that are too large or too small can have detrimental effects. Therefore, careful design and optimization of pore sizes are essential for developing effective scaffolds for tissue engineering applications. Our results demonstrated that our nozzleless centrifugal spinning technique can easily produce the 3D fiber constructs with larger pores than the electrospinning equipment, typically up to the size of tens of micrometers (see Figure 3, Figure 4, Figure 5, Figure 6 and Figure 7).

### 4.3. Effect of Micro/Nanofiber Surface Roughness on Cell Colonization

The combined results of SEM and BET analyses revealed the internal hierarchical porosity of the produced polymeric micro/nanofibers produced by the centrifugal spinning (see Figure 7, Figure 8 and Figure 9 and Table 3), which can influence the surface roughness of the fibers, which is usually in the submicrometer or nanoscale range. PLA fibers with increased nanoscale surface roughness and porosity showed a trend towards higher cell attachment and proliferation [23]. The roughness of nanofibrous scaffolds significantly influences cell attachment, which is a critical factor in tissue engineering applications. Surface roughness can affect cellular behavior such as adhesion, proliferation, and differentiation, ultimately impacting the effectiveness of the scaffold in promoting tissue regeneration. Nanofibrous scaffolds with increased nanoscale surface roughness have been shown to improve cell adhesion. For instance, Zhang et al. [66] reported that the incorporation of β-tricalcium phosphate (β-TCP) nanoparticles into gelatin nanofibers resulted in a rough surface that promoted osteoblast adhesion and proliferation, as well as osteogenic differentiation. This nanoscale roughness provides more surface area for cells to anchor, facilitating the formation of focal adhesions, which are critical for cell signaling and communication with the ECM [67]. Similarly, Liu et al. [68] demonstrated that the regulated surface morphology of polyaniline/polylactic acid composite nanofibers improved biocompatibility and cell adhesion due to the increased surface area provided by the rough texture. Moreover, nanofiber roughness can influence the spatial organization of cells. Gittens et al. [69] highlighted that nanoscale surface features can significantly affect osteoblast behavior, including adhesion and differentiation, suggesting that specific roughness patterns can guide cellular responses. This is particularly relevant in applications where directional growth is desired, such as nerve regeneration, where aligned and rough nanofibers can guide cell migration and orientation [70].

In addition to promoting adhesion, rough surfaces can also enhance the retention of bioactive molecules, which can further stimulate cell attachment and proliferation. For example, Oktay et al. [71] found that collagen-modified nanofibrous scaffolds exhibited improved cell attachment and growth rates, which they attributed to the roughness facilitating better cellular interactions with collagen. The increased surface area allows for the more effective binding of growth factors and ECM proteins, which are essential for cell survival and function. On surfaces with nanoscale roughness, the cell adhesion-mediating molecules are adsorbed in an advantageous, near-physiological and flexible conformation, which facilitates the binding of the active sites in these molecules, e.g., specific amino acid sequences such as RGD, by cell adhesion receptors, such as integrins (for a review, see [1]).

However, it is important to note that while increased roughness may enhance cell attachment, excessively rough surfaces may inhibit cell spreading and migration. For example, Dubey et al. [72] observed that vascular endothelial cells performed better on smoother surfaces compared to rough electrospun surfaces, suggesting that there is an optimal range of roughness that balances adhesion and cell mobility. Nanoscale surface roughness typically promotes cell attachment, spreading and subsequent proliferation, whereas microscale surface roughness may inhibit these processes. This is because mammalian cells typically spread over tens of micrometers; thus, microscale irregularities can interfere with this process. Specifically, cells must adhere to depressions between protrusions that limit their spreading area, or they must bridge these protrusions, thereby not using the entire ventral surface of their cell membrane to interact with their adhesion substrate. If they adhere to both protrusions and depressions, they must bend unphysiologically, mechanically stressing their cell membrane. As a result, the cells proliferate more slowly, although they may achieve a higher degree of differentiation, such as osteogenic differentiation (for a review, see [1]). Therefore, careful design and optimization of nanofiber roughness are necessary to achieve the desired cellular responses.

In summary, the roughness of nanofibrous scaffolds plays a pivotal role in influencing cell attachment. By increasing surface area and promoting favorable interactions with cells, rough nanofibers can significantly improve adhesion, proliferation, and differentiation, making them valuable in tissue engineering applications. Future research should focus on optimizing surface roughness to balance cell attachment and mobility for specific tissue engineering needs.

### 4.4. Effect of Internal Porosity of Individual Fibers on Cell Colonization

The combined results of SEM and BET analyses revealed the internal hierarchical porosity of the polymeric micro/nanofibers produced by the centrifugal spinning (see Figure 7, Figure 8 and Figure 9 and Table 3). The internal structure of nanofibers is characterized by a combination of molecular orientation, component distribution, and the presence of additional materials, all of which contribute to their overall performance. The ability to manipulate these internal structures through various fabrication techniques opens up new avenues for enhancing the functionality of nanofibers in advanced applications. Based on the internal structure, nanofibers are classified into non-porous, mesoporous, hollow and core-shell fibers [73]. Nanofibers can exhibit hierarchical internal porosity, which is a significant advance in materials science, particularly for applications in catalysis, drug delivery, and filtration. Hierarchical porosity refers to the presence of pores at multiple scales within the nanofibers, which enhances their functionality by increasing surface area and providing pathways for mass transport [68,74]. For example, Chen et al. [75] described coaxial porous nanofibers formed through a non-solvent-induced phase separation, resulting in uniform small pores conducive to sustained drug release applications.

Controlling the porosity of nanofibers is crucial for their performance in various applications. For example, Abolhasani et al. [76] discussed methods of hierarchically engineering porosity within nanofibers, emphasizing the importance of controlling thermodynamic and kinetic parameters during fabrication to achieve desired pore structures. Moreover, the use of sacrificial materials and phase separation techniques has been shown to facilitate the creation of complex porous architectures, as evidenced by the work of Gulfam et al. [77], who reported the selective removal of core materials to create hollow or porous structures in polymeric fibers.

In terms of practical applications, the hierarchical porosity of nanofibers significantly enhances their performance. In addition, the high porosity of nanofibers can improve their ability to absorb exudates in wound dressings, as shown by Serag et al. [78], who found that increased porosity allowed for better nutrient exchange and fluid management at wound sites. Furthermore, the synthesis of porous nanofibers can be tailored to meet specific functional requirements. For example, You et al. reported cigar-shaped TiO_2_ nanofibers with hierarchical internal structures that enhanced their mechanical and electrochemical performance, demonstrating the versatility of hierarchical porosity in energy storage applications [79]. Similarly, the work of Nathani and Sharma [80] showed that the sensitivity of biosensors could be significantly improved by optimizing the porosity of electrospun nanofibers. The ability to create nanofibers with hierarchical internal porosity opens up numerous possibilities for advanced materials applications. The combination of different fabrication techniques allows for the precise control of pore structures, which can be tailored to improve the performance of nanofibers in areas such as catalysis, drug delivery and environmental remediation.

The internal porosity of polymeric nanofibers can play a critical role in tissue engineering applications by influencing several key factors, including cell attachment, nutrient transport, and mechanical properties of the scaffolds. The hierarchical porosity within these nanofibers enhances their performance as scaffolds for tissue regeneration, making them highly suitable for mimicking the ECM of native tissues. First, the presence of internal porosity significantly increases the surface area of the nanofibers, which is essential for promoting cell attachment and proliferation. As highlighted by Dahlin et al. [6], the large surface area provided by electrospun nanofibers facilitates better interaction with cells and bioactive factors, thereby enhancing cellular activities crucial for tissue engineering. Moreover, the porous structure allows for improved nutrient and waste transport, which is essential for maintaining cell viability and function within the scaffolds. Kalaoglu-Altan et al. [81] found that the three-dimensional, porous scaffold architecture of polymeric nanofibers provides excellent properties for cell growth and biomolecular immobilization, which are critical for effective tissue regeneration. In addition, the mechanical properties of nanofibers can be tailored by manipulating their porosity. Linh et al. [82] discussed how the degradation behavior of polyvinyl alcohol/gelatin nanofiber composites is influenced by their inner structure, which in turn affects their mechanical integrity and suitability as biodegradable scaffolds. The ability to control porosity allows for the optimization of stiffness and flexibility, ensuring that the scaffolds can withstand physiological loads while providing the necessary support for tissue growth.

Furthermore, the hierarchical porosity can be engineered to create specific microenvironments that promote desired cellular responses. For example, the work of Wang et al. [83] highlights the importance of creating biomimetic structures that not only mimic the physiological properties of the ECM but also provide biochemical cues for cell differentiation and tissue formation. The incorporation of growth factors or other bioactive molecules into the porous structure can further enhance the functionality of the scaffolds, as demonstrated by Oliveira et al. [84], who investigated the binding of growth factors to nanofibrous substrates to promote fibroblast activity. Moreover, the degradation rate of the nanofibers, which is critical for tissue engineering applications, can also be influenced by their porosity. As noted by Priyanto et al. [85], the porosity of nanofibers affects their interaction with body fluids, thereby influencing their degradation behavior and the release of encapsulated therapeutic agents. This controlled degradation is essential for scaffolds to provide temporary support while allowing for the gradual replacement by natural tissue.

Therefore, the influence of nanoporosity on water retention in tissue engineering scaffolds is a critical factor affecting the performance and functionality of these materials. Nanoporosity increases the ability of scaffolds to retain water, which is essential for maintaining a suitable environment for cell growth and tissue regeneration. The relationship between nanoporosity and water retention can be understood through several mechanisms, including increased surface area, improved hydrophilicity, and enhanced nutrient diffusion. First, the presence of nanoporosity significantly increases the specific surface area of scaffolds, which allows for greater water retention. Depan and Misra [86] emphasize that scaffolds with nanostructured features exhibit favorable water retention behavior, which is conducive to cell adhesion and proliferation. This is particularly important in tissue engineering, where the scaffolds must provide a supportive environment for cell growth. The retained moisture not only promotes cell adhesion but also enhances signal-transduction pathways that are crucial for cellular activities.

Moreover, the hydrophilicity of scaffolds is often enhanced by their nanostructured surfaces. For instance, Ghalei et al. [87] found that electrospun scaffolds with increased wettability allow for better cell attachment, which is directly related to improved water retention capabilities. This wettability is influenced by the size and distribution of nanopores, which can trap water molecules and create a favorable microenvironment for cells. Additionally, the study by Li et al. [88] highlights that hydrogels with high water content are essential for effective wound healing, further emphasizing the importance of water retention in tissue engineering applications. Capillary action plays an important role in the water retention capacity of tissue engineering scaffolds. The interconnected pores created by nanoporosity facilitate the movement of water through the scaffold, allowing for effective nutrient diffusion and waste removal. Zhao et al. [89] highlighted that the ability of scaffolds to absorb and retain fluids is critical for maintaining a hydrated wound bed, which is essential for skin regeneration. The capillary forces generated by the porous structure allow the scaffolds to draw water into their matrix, thereby improving hydration and nutrient availability to cells.

The internal porosity of polymeric nanofibers is a critical factor in their application for tissue engineering. It enhances cell attachment and proliferation, optimizes mechanical properties, and allows for the controlled release of bioactive substances, all of which contribute to effective tissue regeneration. The ability to engineer these porous structures opens up new avenues for the development of advanced scaffolds tailored to specific tissue engineering needs. Both nanoporosity and capillary effects are fundamental to the water retention capabilities of tissue engineering scaffolds. The increased surface area provided by nanoporosity enhances water retention, while capillary action facilitates the movement of water and nutrients within the scaffolds.

Taken together, all these factors help to create a suitable environment for cell growth and tissue regeneration, making them essential considerations in the design of scaffold for tissue engineering applications. For these purposes, we tested the scaffolds prepared from single polymers (PLA, PHB, PBS, and PCL) and several copolymers, namely, PLA/PHB 4/1; PHB/PBS 1/4, 2/7; and PLA/PCL 5/1, 13.5/4 (*w*/*w*). The results of our study showed that the PLA/PCL 13.5/4 scaffolds appeared to be the most advantageous as they had the deepest penetration of ASCs. This can be attributed to the most favorable parameters promoting cell ingrowth inside the scaffolds, the largest macropore size, the largest nanopore volume, and the largest specific surface area of the PLA/PCL 13.5/4 scaffolds.

Finally, our fibrous scaffolds were designed to special fat substitutes, i.e., very ductile, soft, and flexible. However, nanofibrous scaffolds have emerged as a pivotal component in various fields of tissue engineering due to their unique properties that closely mimic the natural ECM. These scaffolds are characterized by their high surface area, porosity, and ability to support cell adhesion and proliferation, making them suitable for a range of applications including bone, skin, and cardiac tissue engineering. In bone tissue engineering, nanofibrous scaffolds are particularly advantageous because they can mimic the hierarchical structure of natural bone. Several methods have been developed to improve the mechanical properties of the nanofibrous scaffolds, including the incorporation of reinforcing materials, structural modifications, and advanced fabrication techniques [90]. Incorporation of bioactive molecules into nanofibers for tissue engineering is a promising strategy to enhance the biological functionality of scaffolds. This approach can significantly improve cell adhesion, proliferation, and differentiation, ultimately leading to better tissue regeneration outcomes. Several methods have been developed to achieve this integration, each with its unique advantages and applications (for a review, see [91]).

## 5. Conclusions and Further Perspectives

The aim of our study was to verify the feasibility of using our own patented nozzle-free centrifugal technology to produce micro/nanofibrous scaffolds from various biocompatible and biodegradable polymers.

We found that single polymers, such as PLA, PHB, PBS, and PCL, formed a relatively dense network of micro/nanofibers that were unsuitable for cell colonization due to small pore sizes, typically on the order of units of micrometers, but exceptionally up to tens of micrometers. Similarly, PLA/PHB, PHB/PCL, and PHB/PBS blends again formed relatively dense networks with pore sizes in the order of units up to tens of micrometers, regardless of the ratio of individual polymers in the blends. The largest pores (in tens of micrometers) were obtained with the PLA/PCL blends, regardless of the tested ratios (5/1; 13.5/4 *w*/*w*) of both polymers.

The combined results of SEM and BET analyses revealed the hierarchical porosity of the polymeric micro/nanofibers produced via the centrifugal spinning. In addition to the microporosity of the individual fibers visible in the SEM images (pore size in the order of hundreds of nanometers), the BET analysis revealed internal nanoporosity of the fibers, with pore size in the range of 1–14 nm. The addition of PLA to PCL resulted in a significant increase in both the specific surface area and BJH pore size (2–20 nm). The hierarchical porosity of our polymeric micro/nanofibrous scaffolds can significantly influence their water retention capacity and fiber roughness, which are important for cell colonization of the scaffolds and their applicability in tissue engineering.

Based on the relatively large pore size of the fiber networks, high internal porosity, and large specific surface area of the 3D fiber constructs, we evaluated the sample of PLA/PCL 13.5/4 (*w*/*w*) blend sample as the material with the most favorable properties for cell colonization. Measurement of the thickness of the layer into which cells migrated on different types of scaffolds confirmed that the PLA/PCL 13.5/4 (*w*/*w*) fibrous scaffolds showed the deepest ASC ingrowth.

We therefore created a bioartificial adipose tissue substitute using 3D micro/nanofibrous scaffolds in combination with ASCs, which are precursors of adipocytes, capable of differentiating into this cell type. These adipose tissue analogues could be used in the future instead of classical fat grafts, applied, e.g., for breast reconstruction after a cancer ablation procedure. We expect that our cell–material constructs could reduce post-operative atrophy.

## Figures and Tables

**Table 1:** Parameters of the centrifugal spinning (CF) using the Nanocent demonstrator.

Spinned Solution in CF	PLA	PHB	PBS	PCL	PHB/PLA	PHB/PBS	PLA/PCL
Polymers weight ratio (*w*/*w*)					4/1	1/4, 2/7	5/1, 13.5/4
Total dry matter % (*w*/*w*) in solutions	10.00	2.00	14.00	10.00	2.38	6.36, 6.00	10.00
Rotation speed (rpm)	3500	3000	4000	3000	3500	3500	1500–5000
Solution flow (mL/min)	15	8	7	8	10	12	15
Air flow (m^3^/h)	60–65	55–60	25–30	8	10	66–69	15

**Table 2:** Influence of rotary speed on the PLA/PCL 13.5/4 fiber diameter.

Rottary Speed of the Spinning Disc (rpm)	Median of Fiber Diameter (µm)
1500	6.84 ± 5.77
3000	5.71 ± 3.61
5000	7.81 ± 3.79

**Table 3:** Results of BET analysis of the 3D fibrous scaffolds.

SAMPLE	Specific Surface Area (m^2^/g)	BJH Pore Size (cm^3^/g)
	AM ± SD	AM ± SD
PHB (not sterilized)	12.4 ± 2.6	0.013 ± 0.003
PHB (sterilized)	15.2 ± 1.7	0.018 ± 0.005
PLA (not sterilized)	15.2 ± 1.9	0.014 ± 0.002
PLA (sterilized)	15.5 ± 1.6	0.011 ± 0.003
PLA/PCL 5/1 (sterilized)	36.1 ± 2.0	0.045 ± 0.004
PLA/PCL 13.5/4 (sterilized)	63.9 ± 17.6	0.063 ± 0.003

**Table 4:** The thickness of the layer into which cells migrated on different types of scaffolds. Mean ± S.D. of two–three samples for each experimental group (ANOVA, Student–Newman–Keuls method).

SAMPLE	Cell Ingrowth (µm)		
	Day 2	Day 6	Day 10
PLA	171 ± 4	141 ± 42	153 ± 14
PHB	154 ± 20	104 ± 23	116 ± 38
PHB/PBS 1/4 (*w*/*w*)	n.a.	180 ± 42	175 ± 35
PHB/PBS 2/7 (*w*/*w*)	171 ± 1	175 ± 20	173 ± 18
PLA/PHB 4/1 (*w*/*w*)	153 ± 10	162 ± 20	158 ± 50
PLA/PCL 5/1 (*w*/*w*)	160 ± 42	147 ± 37	241 ± 36
PLA/PCL 13.5/4 (*w*/*w*)	202 ± 64	427 ± 98	413 ± 66

## Data Availability

The data presented in this study are available on request from the corresponding author.

## Supplementary Materials

The following supporting information can be downloaded at: https://www.mdpi.com/article/10.3390/polym17030386/s1, Figure S1: Lipid droplet formation in ASCs in 12-day-old cultures in DMEM with 10% of FBS (A), DMEM + FBS + dexamethasone + indomethacin + insulin (B), DMEM + FBS + dexamethasone + indomethacin + insulin + 0.5 mM IBMX (C) and in DMEM + rabbit serum + dexamethasone + indomethacin + insulin (D). Lipid droplets stained with Oil Red O, cells counterstained with hematoxylin. Olympus IX 51 microscope, obj. 20x, DP 74 digital camera, scale bar 100 μm; Figure S2: Lipid content in cocultures of ASCs and HUVECs after 3, 7, and 14 days of cultivation in six different types of media (1–6), estimated by the absorbance of Oil Red O extracted from the cells. Mean ± S.D. of 2 to 4 measurements for each experimental group and time interval. ANOVA, Student-Newman-Keuls method. Statistical significance (*p* ≤ 0.05): x, #: compared with the value on days 3 and 7, respectively; all: compared with all samples on the indicated day; 1, 2: compared with the corresponding values in media 1 and 2, respectively; Figure S3: Oil Red O staining of ASCs and HUVECs in cocultures after 3 days of exposure to six different types of culture media. A: EGM-2 (medium 1), B: EGM-2 + DMEM + FBS (medium 2), C: EGM-2 + DMEM + FBS + dexamethasone + indomethacin + insulin (medium 3), D: EGM-2 + DMEM + FBS + dexamethasone + indomethacin + insulin + 0.25 mM IBMX (medium 4), E: EGM-2 + DMEM + FBS + dexamethasone + indomethacin + insulin + 0.5 mM IBMX (medium 5), F: EGM-2 + DMEM + rabbit serum + dexamethasone + indomethacin + insulin (medium 6). Olympus IX 71 microscope, obj. 20x, DP 80 digital camera, scale bar 50 μm; Figure S4: Oil Red O staining of ASCs and HUVECs in cocultures after 14 days of exposure to six different types of culture media. A: EGM-2 (medium 1), B: EGM-2 + DMEM + FBS (medium 2), C: EGM-2 + DMEM + FBS + dexamethasone + indomethacin + insulin (medium 3), D: EGM-2 + DMEM + FBS +dexamethasone + indomethacin + insulin + 0.25 mM IBMX (medium 4), E: EGM-2 + DMEM + FBS + dexamethasone + indomethacin + insulin + 0.5 mM IBMX (medium 5), F: EGM-2 + DMEM + rabbit serum + dexamethasone + indomethacin + insulin (medium 6). Olympus IX 71 microscope, obj. 20x, DP 80 digital camera, scale bar 50 μm; Figure S5: Coculture of ASCs and HUVECs on day 3 after seeding in EGM-2 medium. HUVECs are labeled in red with CellTracker™ Red CMTPX. Olympus IX 71 microscope, obj. 10x, DP 80 digital camera, scale bar 100 μm; Figure S6: Cocultures of ASCs and HUVECs after 4 and 7 days of exposure to six different types of media (1–6). HUVECs are labeled in red with CellTracker™ Red CMTPX. Olympus IX 71 microscope, obj. 10x, DP 80 digital camera, scale bars 100 μm. Figure S7: Cell population density in cocultures of ASCs and HUVECs after 3, 7, and 14 days of cultivation in six different types of media (1–6), estimated by counting DAPI-stained cell nuclei. Mean ± S.D. of 2 to 4 cell samples for each experimental group and time interval. ANOVA, Student-Newman-Keuls method. Statistical significance (*p* ≤ 0.05): x, #: compared with the value on days 3 and 7, respectively; all: compared with all samples on the indicated day; Figure S8: FTIR spectra blended PLA/PCL (weight ratio 5/1 and 13,5/4) fibers. Fourier-transform infrared (FTIR) spectrometer Nicolet iS5 (Fisher Scientific, Waltham, MA, USA) with a diamond crystal iD7 ATR accessory; Figure S9: Designation of functional groups of the PLA and PCL polymers on FTIR spectrum a blended PLA and PCL material (help to identify the functional groups in the previous figure); Figure S10: Scheme of the demonstrator NANOCENT for nozzleless centrifugal spinning; Figure S11: Histograms of PLA/PCL 13,5/4 fiber diameters obtained by the centrifugal spinning at different rotation speed.
