# Supplementary material for: Evaluation of Polymeric Micro/Nanofibrous Hybrid Scaffolds Prepared via Centrifugal Nozzleless Spinning for Tissue Engineering Applications
Source: Polymers (Basel). 2025 Jan 31;17(3):386. doi: 10.3390/polym17030386 (PMC11820018; doi:10.3390/polym17030386)
Supplement: Supplementary file 1 [file polymers-17-00386-s001.zip › polymers-3414855-supplementary.pdf]

Supplementary Materials

Evaluation of polymeric micro/nanofibrous hybrid scaffolds prepared by centrifugal nozzleless spinning for tissue engineering applications

Miloš Beran[1*], Jana Musílková[2*], Antonín Sedlář[2], Petr Slepička[3], Martin Veselý[3], Zdeňka Kolská[4], Ondřej Vltavský[1], Martin Molitor[5], Lucie Bačáková[2]

[1] Food Research Institute in Prague, Radiova 1285/7, 102 00 Prague 10 - Hostivar, Czech Republic

[2] Institute of Physiology of the Czech Academy of Sciences, Videnska 1083, 142 00 Praha 4, Czech Republic

[3] University of Chemistry and Technology Prague, Technicka 5, 166 28 Prague 6-Dejvice, Czech Republic

[4] J. E. Purkyne University in Usti nad Labem, Pasteurova 3544/1, 400 96 Usti nad Labem, Czech Republic

[5] Department of Plastic Surgery, First Faculty of Medicine Charles University and Na Bulovce Hospital, Budinova 67/2, 180 81 Prague 8 - Liben, Czech Republic

*Correspondence: milos.beran@carc.cz; jana.musilkova@fgu.cas.cz

Adipogenic differentiation of adipose-derived stem cells (ASCs)

For adipogenic differentiation, ASCs were seeded in 24-well polystyrene plates (TPP, Trasadingen, Switzerland) at a density of 50,000 cells/well. The cells were cultured in a Dulbecco's modified Eagle's Medium (DMEM; Sigma-Aldrich, MO, USA) supplemented with 10% fetal bovine serum (FBS; Sebak GmbH, Aidenbach, Germany) and gentamicin (40 µg/mL, LEK, Ljubljana, Slovenia) at 37°C in a humidified air atmosphere containing 5% CO_2. After 2 days of cultivation, this medium was changed to one of the following three types of adipogenic media:

(1) DMEM with 10% FBS, 1 µM dexamethasone, 20 µM indomethacin, and 10 µg/mL of insulin,

(2) DMEM with 10% FBS, 1 µM dexamethasone, 20 µM indomethacin, 10 µg/mL of insulin, and 0.5 mM IBMX, or

(3) DMEM with 10 % of rabbit serum (RS; Sigma-Aldrich, Merck, Darmstadt, Germany, Cat. No. R4505), 1 µM dexamethasone, 20 µM indomethacin and 10 µg/mL of insulin.

In medium (1) and (2), the cells were cultured for 12 days, and in medium (3), the cells were cultured for 11 days after one day of preincubation in medium (2).

The cells were then stained with the Lipid (Oil Red O) Staining Kit (Cat. No. MAK194, Sigma-Aldrich, St. Louis, MO, USA) according to the manufacturer's protocol; the cells were counterstained with hematoxylin. The staining revealed that the cells in adipogenic media developed multiple lipid-containing droplets inside the cells, while the formation of these droplets was minimal or even absent in the conventional DMEM medium without adipogenic supplements (**Fig. S1**). The highest

content of lipid-containing droplets was detected in cells cultured in the adipogenic medium supplemented with rabbit serum. Adipogenic differentiation of ASCs and adipose tissue engineering are of great importance for plastic and reconstructive surgery of soft tissue defects caused by trauma, congenital anomalies or surgical resection.

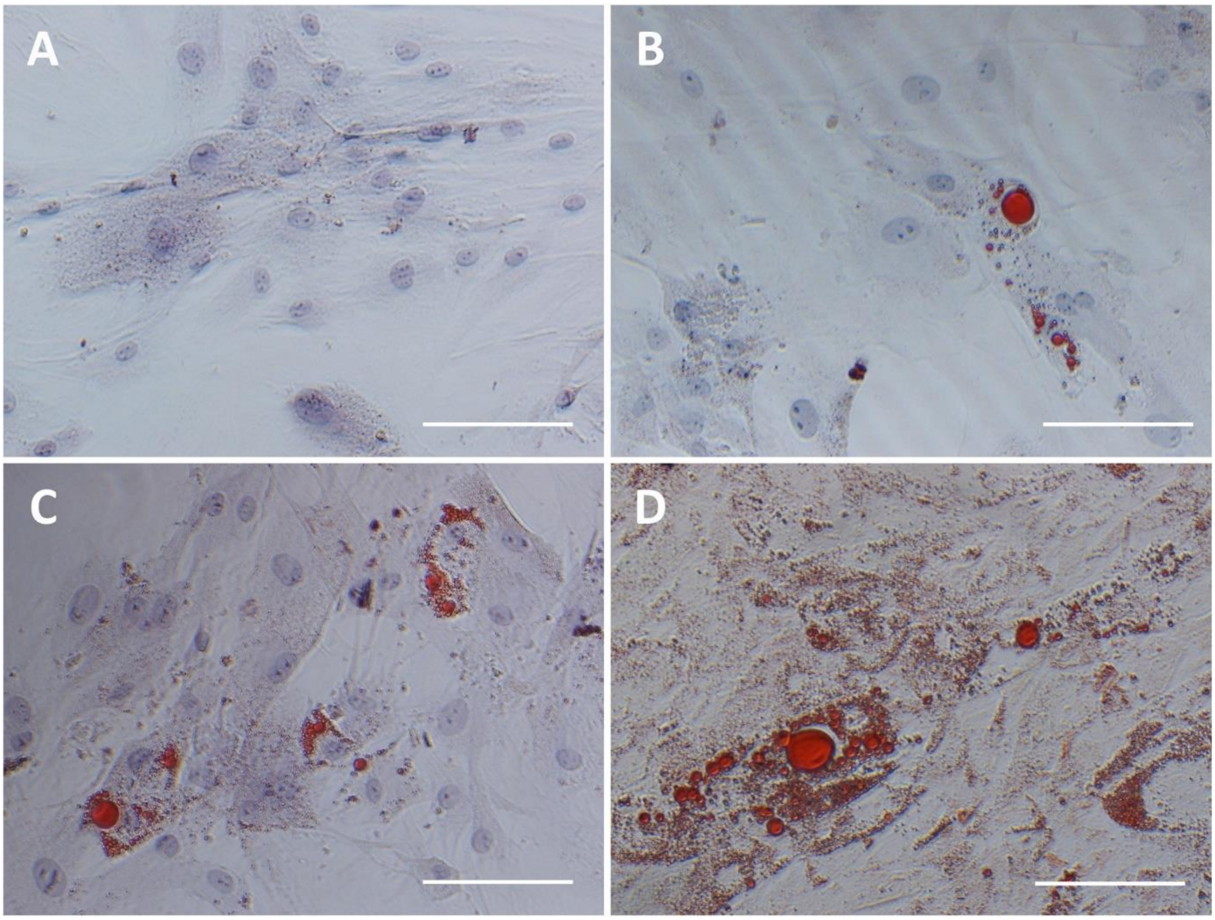

Figure S1. *Lipid droplet formation in ASCs in 12-day-old cultures in DMEM with 10% of FBS (**A**), DMEM + FBS + dexamethasone + indomethacin + insulin (**B**), DMEM + FBS + dexamethasone + indomethacin + insulin + 0.5 mM IBMX (**C**) and in DMEM + rabbit serum + dexamethasone + indomethacin + insulin (**D**). Lipid droplets stained with Oil Red O, cells counterstained with hematoxylin. Olympus IX 51 microscope, obj. 20x, DP 74 digital camera, scale bar 100 µm.*

The adipogenic differentiation of ASCs was also investigated in co-cultures of ASCs with endothelial cells. This was because endothelial cells are required for the vascularization of tissue-engineered constructs, including adipose tissue substitutes, which is necessary for their long-term viability and proper function. Endothelial cells were used in the form of commercially available human umbilical vein endothelial cells (HUVECs; PromoCell, (Heidelberg, Germany, Cat. No. C-12200). The cells were seeded in 24-well polystyrene plates (TPP, Trasadingen, Switzerland) at a ratio of ASCs:HUVECs of 1:2, i.e., 20,000 ASCs and 40,000 HUVECs per well.

For the first three days, the cells were cultured in endothelial cell growth medium 2 (EGM-2), which was prepared from endothelial cell basal medium 2 (EBM-2, PromoCell, Heidelberg, Germany, Cat. No. C-22111), supplemented with the growth medium 2 supplement pack (PromoCell, Heidelberg, Germany, Cat. No. C-39211) containing hydrocortisone, heparin, ascorbic acid, epidermal growth factor (EGF), vascular endothelial growth factor (VEGF), insulin-like growth factor-1 (IGF-1),

FGF-2, 2% of FBS, and also with 1% antibiotic antimycotic solution (v/v, A5955, Sigma-Aldrich, MO, USA).

After three days of cultivation in the EGM-2 medium, the cells were cultured for an additional 3, 7, or 14 days in one of the following six types of media:

(1) EGM-2 medium,

(2) EGM-2 medium (i.e., the medium recommended for HUVECs) mixed with DMEM medium containing 10% FBS (i.e. the medium commonly used for ASCs) at a 1:1 ratio,

(3) EGM-2 medium mixed with DMEM medium (containing 10% FBS) in a 1:1 ratio and further supplemented with factors promoting adipogenic cell differentiation (diff), namely 1 µM dexamethasone, 20 µM indomethacin, and 10 µg/mL of insulin,

(4) EGM-2 medium mixed with DMEM medium (containing 10% FBS) in a 1:1 ratio and further supplemented with 1 µM dexamethasone, 20 µM indomethacin, 10 µg/mL of insulin, and 0.25 mM IBMX,

(5) EGM-2 medium mixed with DMEM medium (containing 10% FBS) in a 1:1 ratio, further supplemented with 1 µM dexamethasone, 20 µM indomethacin, 10 µg/mL of insulin, and 0.5 mM IBMX,

(6) EGM-2 medium mixed with DMEM medium (containing 10% of RS) in a 1:1 ratio, further supplemented with 1 µM dexamethasone, 20 µM indomethacin, and 10 µg/mL of insulin. Prior to cultivation in this medium, the cells were preincubated for one day in medium 5.

Cells were then stained with the Lipid (Oil Red O) Staining Kit (Cat. No. MAK194, Sigma-Aldrich, St. Louis, MO, USA) according to the manufacturer's protocol. After microphotographing the cells, the lipid-staining dye was extracted from the cells in 100% isopropanol (250 µL per well, 15 min incubation). The solution was transferred to 96-well plates (100 µL per well), and the absorbance was measured at 510 nm in Versa Max Microplate Reader (Molecular Devices Corporation, Sunnyvale, California, USA). For each experimental group of cells, 2 to 4 samples were measured.

This quantitative measurement (**Fig. S2**) showed that after 3 and 7 days of adipogenic differentiation, the content of Oil Red O-stained lipids was generally similar in cells in media 1-5. Only in media 4 and 5, i.e. media supplemented with 0.25 mM or 0.5 IBMX, the content of these lipids was slightly but significantly higher than in media 1 and 2, i.e. media without adipogenic supplements. The lipid content of cells grown in media 1-5 reached a statistically significant increase after 14 days of culture. This increase was most pronounced in media 2, 3, 4, i.e. EGM-2 and DMEM 1:1 mixtures, and interestingly, was similar in media without and with osteogenic supplements. The lowest increase in lipid content was obtained for cells in EGM-2 medium (1), and a moderate increase for cells in medium 5 with 0.5 IBMX. Thus, it seems that the lower concentration of IBMX (0.25 mM) had a more favorable effect on adipogenic cell differentiation than the higher concentration (0.5 mM). Interestingly, in the medium 6 containing rabbit serum (RS), the lipid content was clearly and significantly higher than in the cells in the other media already from day 3 of differentiation, and this value was stable until day 14 of differentiation, although this day was surpassed by the cells in the media 2, 3 and 4.

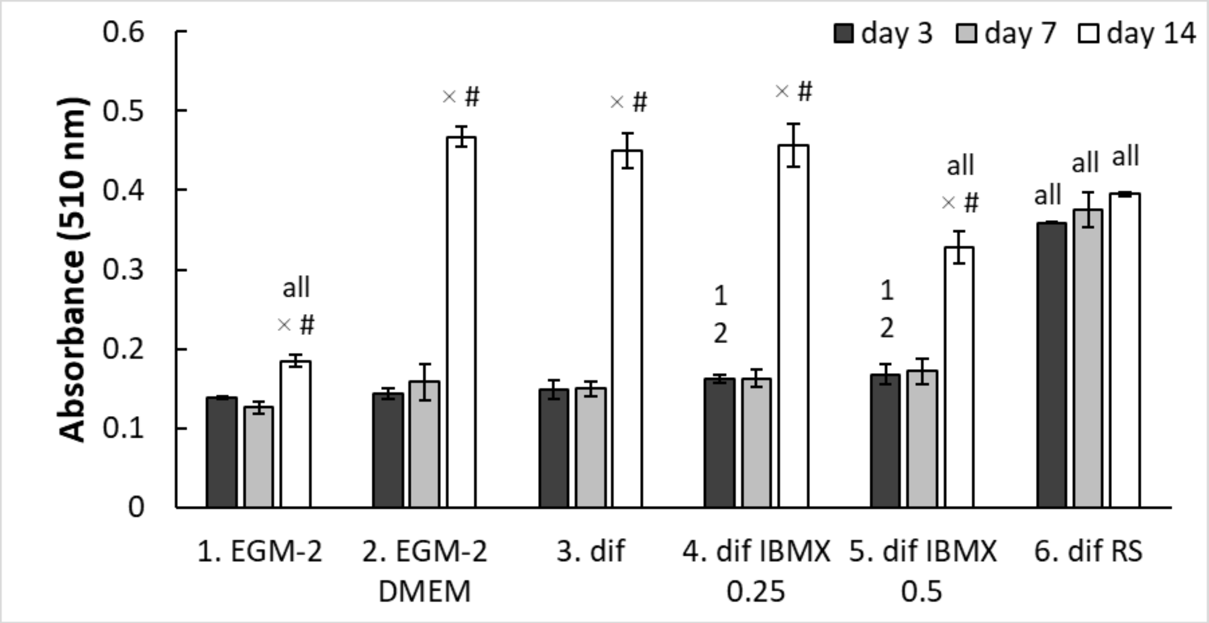

Figure S2. *Lipid content in cocultures of ASCs and HUVECs after 3, 7, and 14 days of cultivation in six different types of media (1-6), estimated by the absorbance of Oil Red O extracted from the cells. Mean ± S.D. of 2 to 4 measurements for each experimental group and time interval. ANOVA, Student-Newman-Keuls method. Statistical significance ($p \leq 0.05$): x, #: compared with the value on days 3 and 7, respectively; all: compared with all samples on the indicated day; 1, 2: compared with the corresponding values in media 1 and 2, respectively.*

These quantitative results were also supported by qualitative results, i.e. staining of cells in micrographs (**Fig. S3**). After three days of adipogenic differentiation, the most intense Oil Red O staining was obtained in cells grown in rabbit serum (RS) medium (6), although reddish staining is also well visible in cells in media 4 and 5, i.e., with IBMX.

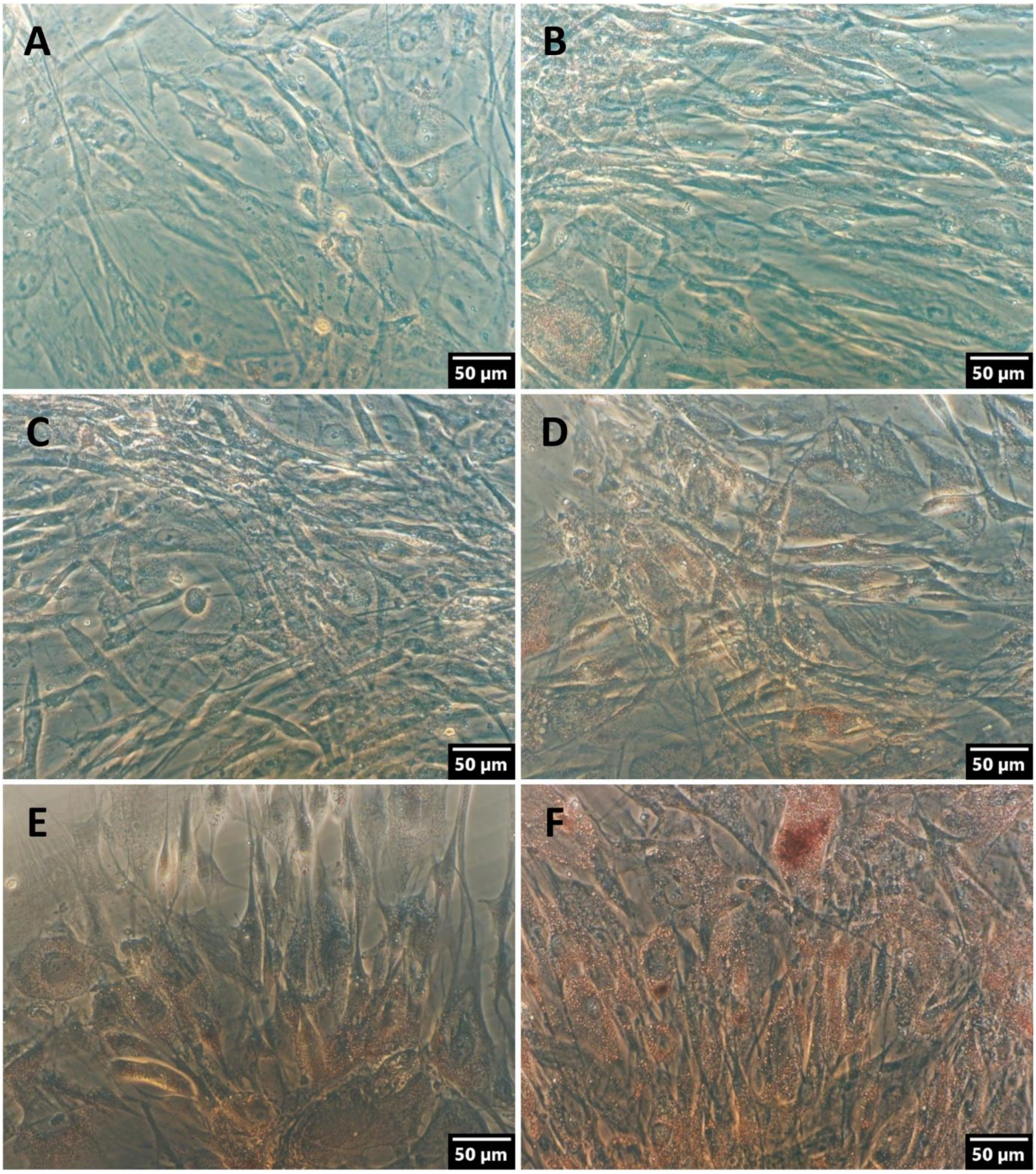

Figure S3. *Oil Red O staining of ASCs and HUVECs in cocultures after 3 days of exposure to six different types of culture media.* ***A***: *EGM-2 (medium 1),* ***B***: *EGM-2 + DMEM + FBS (medium 2),* ***C***: *EGM-2 + DMEM + FBS + dexamethasone + indomethacin + insulin (medium 3),* ***D***: *EGM-2 + DMEM + FBS + dexamethasone + indomethacin + insulin + 0.25 mM IBMX (medium 4),* ***E***: *EGM-2 + DMEM + FBS + dexamethasone + indomethacin + insulin + 0.5 mM IBMX (medium 5),* ***F***: *EGM-2 + DMEM + rabbit serum + dexamethasone + indomethacin + insulin (medium 6). Olympus IX 71 microscope, obj. 20x, DP 80 digital camera, scale bar 50 µm.*

After 14 days of adipogenic differentiation, cells in all media except EGM-2 were intensely stained with Oil Red O, and this intensity was particularly high in cells in medium 6 with rabbit serum. Some cells in IBMX media (media 4 and 5) formed large, clearly distinguishable lipid droplets, whereas the

droplets in cells in the other media were smaller and were homogeneously distributed throughout the cell cultures (**Fig. S4**).

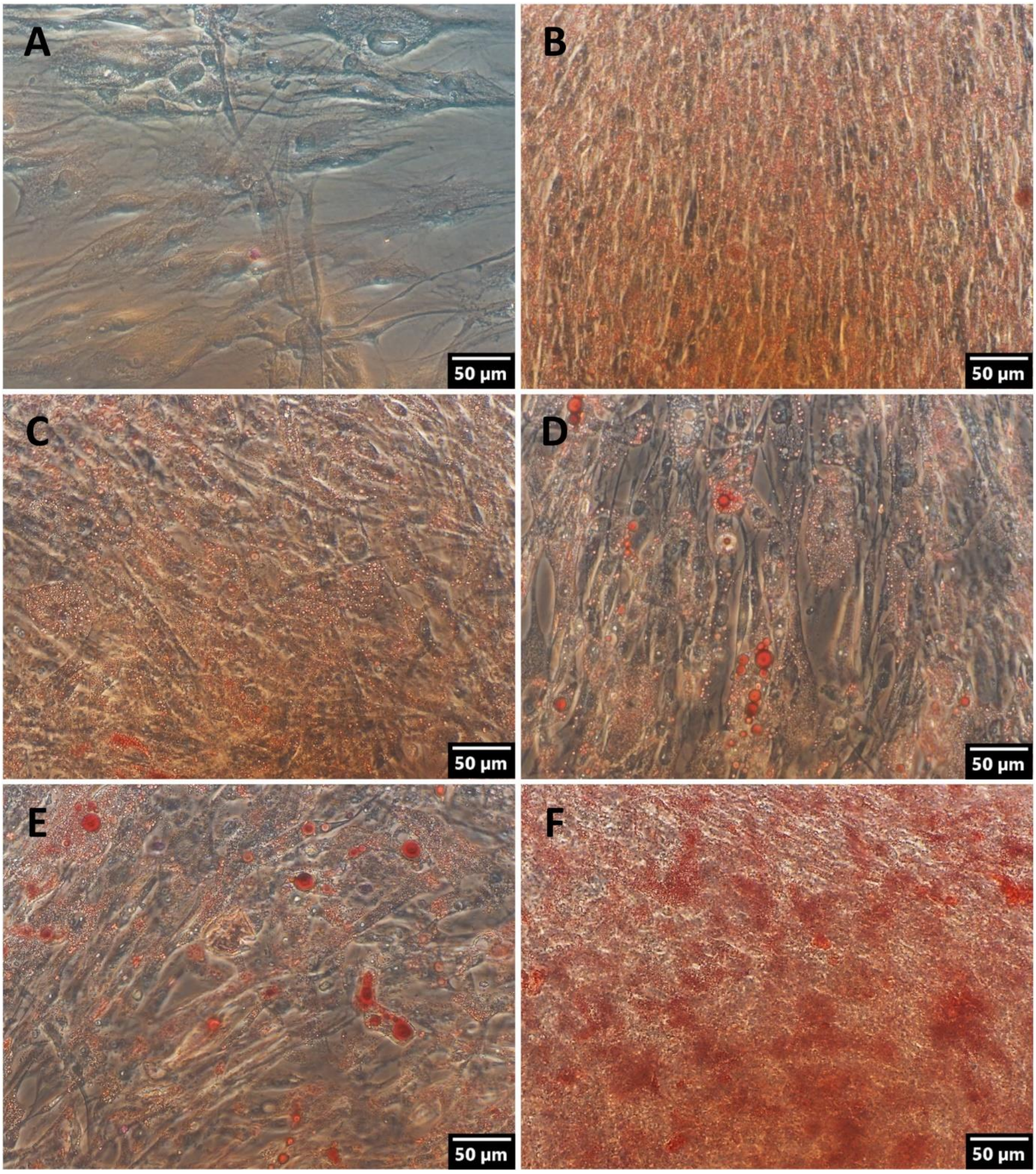

Figure S4. *Oil Red O staining of ASCs and HUVECs in cocultures after 14 days of exposure to six different types of culture media.* ***A****: EGM-2 (medium 1),* ***B****: EGM-2 + DMEM + FBS (medium 2),* ***C****: EGM-2 + DMEM + FBS + dexamethasone + indomethacin + insulin (medium 3),* ***D****: EGM-2 + DMEM + FBS + dexamethasone + indomethacin + insulin + 0.25 mM IBMX (medium 4),* ***E****: EGM-2 + DMEM + FBS + dexamethasone + indomethacin + insulin + 0.5 mM IBMX (medium 5),* ***F****: EGM-2 + DMEM + rabbit serum + dexamethasone + indomethacin + insulin (medium 6). Olympus IX 71 microscope, obj. 20x, DP 80 digital camera, scale bar 50 µm.*

To distinguish ASCs and endothelial cells in their co-cultures, HUVECs were labeled with CellTracker™ Red CMTPX (Thermo Fisher Scientific, Waltham, MA, USA, Cat. No. C34552, 18 µM concentration in EGM-2 medium, incubation 30 min). After 3 days of cultivation in EGM-2 medium, i.e. before changing the medium to media 1 to 6, both cell types were present in the cultures, were viable and had a physiological morphology (**Fig. S5**), and this picture was also maintained at later culture intervals, i.e. after an additional 4 or 7 days of exposure to six different media types (1 to 6, **Fig. S6**).

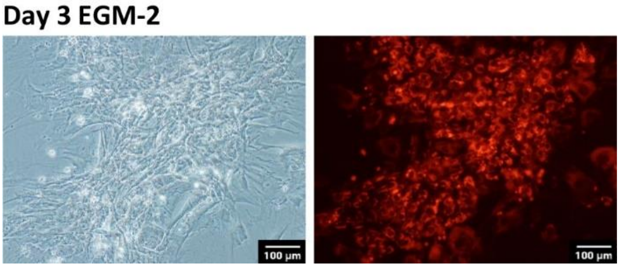

Figure S5*. Coculture of ASCs and HUVECs on day 3 after seeding in EGM-2 medium. HUVECs are labeled in red with CellTracker™ Red CMTPX. Olympus IX 71 microscope, obj. 10x, DP 80 digital camera, scale bar 100 µm.*

Day 7 (day 4 of differentiation)

1. EGM-2

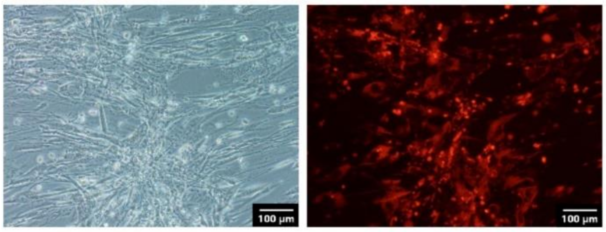

2. DMEM + EGM-2

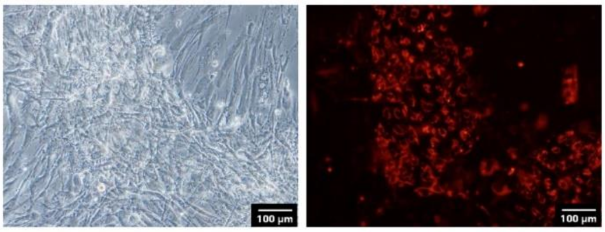

3. diff

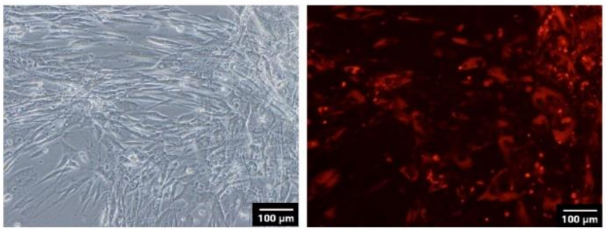

4. diff + IBMX 0.25

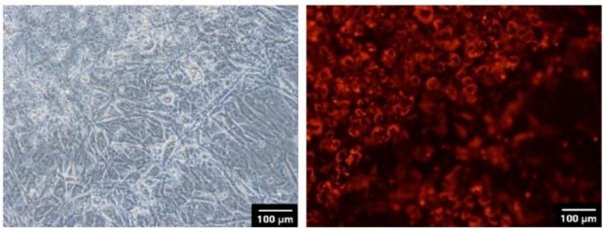

5. diff + IBMX 0.5

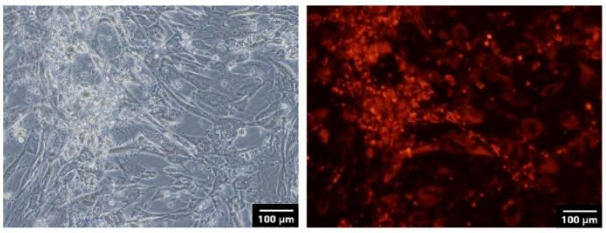

6. diff + RS

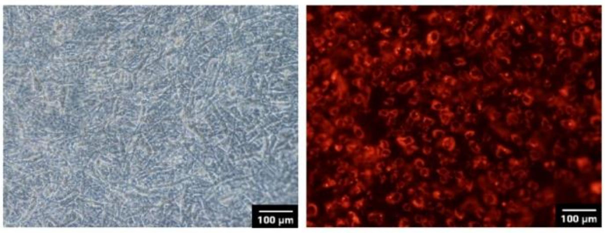

Day 10 (day 7 of differentiation)

1. EGM-2

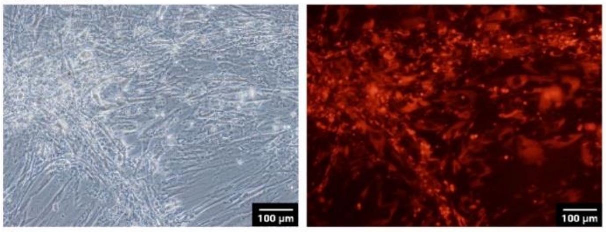

2. DMEM + EGM-2

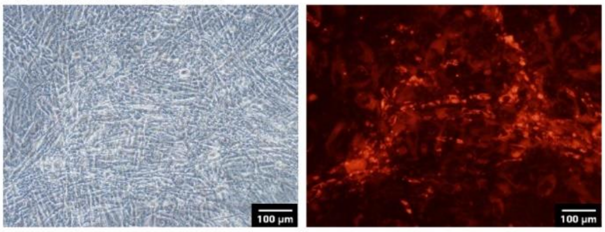

3. diff

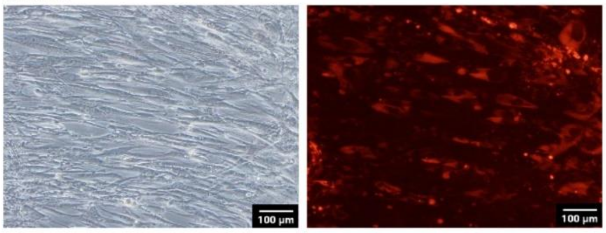

4. diff + IBMX 0.25

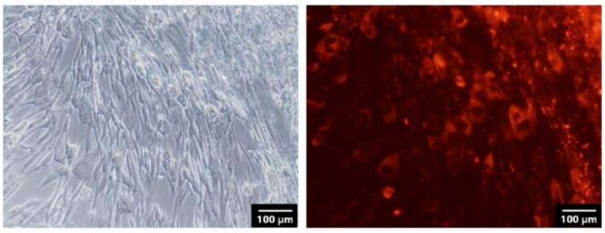

5. diff + IBMX 0.5

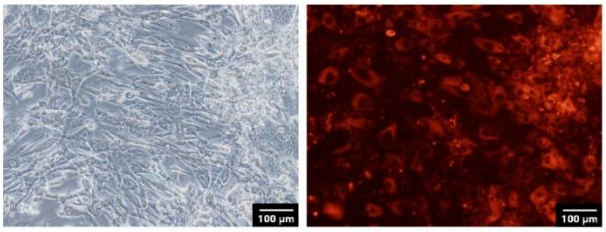

6. diff + RS

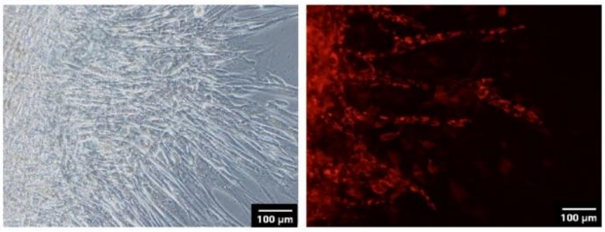

Figure S6. *Cocultures of ASCs and HUVECs after 4 and 7 days of exposure to six different types of media (1-6). HUVECs are labeled in red with CellTracker™ Red CMTPX. Olympus IX 71 microscope, obj. 10x, DP 80 digital camera, scale bars 100 μm.*

Counting of cell nuclei counterstained with 4',6-diamidin-2-fenylindol (DAPI; Sigma-Aldrich, Cat. No. 32670, 20 μg/mL) showed that cells grew well in all media tested except EGM-2, where cell numbers stagnated from day 3 to 14 (**Fig. S7**). The best cell growth (i.e. the highest final cell population density) was obtained in medium 2, i.e. the 1:1 mixture of EGM-2 and DMEM, whereas in the same mixture with supplements promoting adipogenic cell differentiation, the cell proliferation activity was lower. Interestingly, in medium 6, where DMEM was supplemented with rabbit serum instead of FBS, the cells (in addition to high adipogenic differentiation) also reached a relatively high final cell population density.

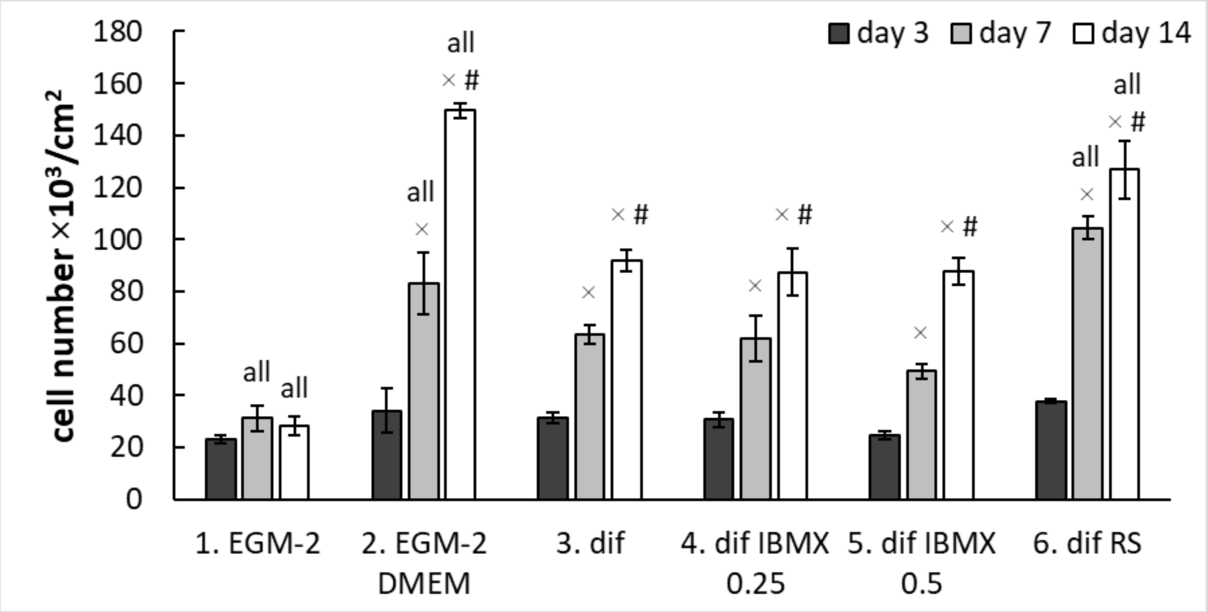

Figure S7. *Cell population density in cocultures of ASCs and HUVECs after 3, 7, and 14 days of cultivation in six different types of media (1-6), estimated by counting DAPI-stained cell nuclei. Mean ± S.D. of 2 to 4 cell samples for each experimental group and time interval. ANOVA, Student-Newman-Keuls method. Statistical significance ($p \leq 0.05$): x, #: compared with the value on days 3 and 7, respectively; all: compared with all samples on the indicated day.*

To prove the absence of phase separation in the PLA and PCL blends we used FTIR. In the case of PLA/PCL blends, the identification of phase separation can be inferred from the shifts in characteristic absorption bands associated with the functional groups of each polymer. We have not observed any such shifts.

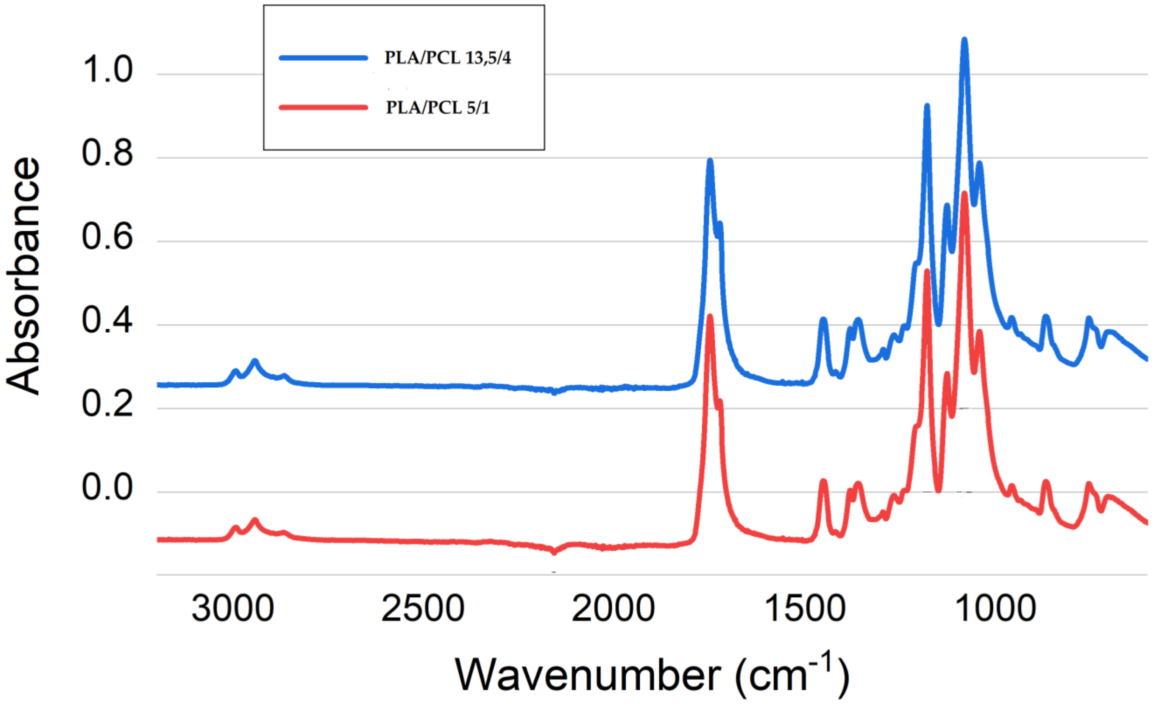

Figure S8. *FTIR spectra blended PLA/PCL (weight ratio 5/1 and 13,5/4) fibers. Fourier-transform infrared (FTIR) spectrometer Nicolet iS5 (Fisher Scientific, Waltham, MA, USA) with a diamond crystal iD7 ATR accessory.*

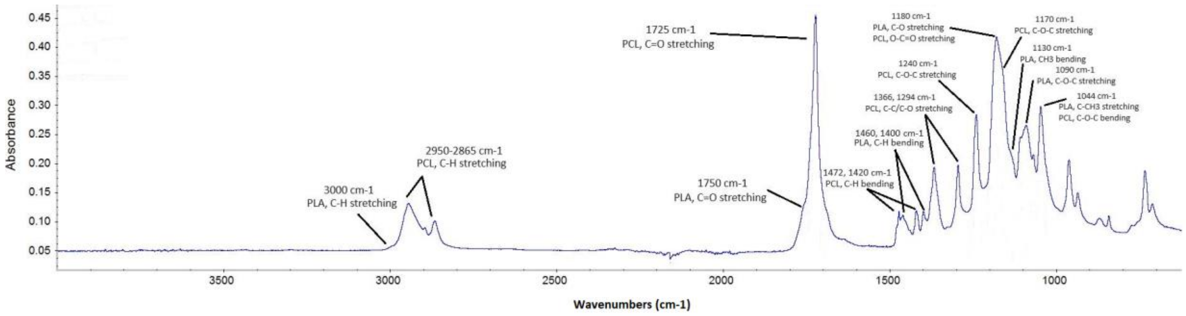

Figure S9. *Designation of functional groups of the PLA and PCL polymers on FTIR spectrum a blended PLA and PCL material (help to identify the functional groups in the previous figure).*

Figure S10 shows scheme of the demonstrator NANOCENT for nozzleless centrifugal spinning.

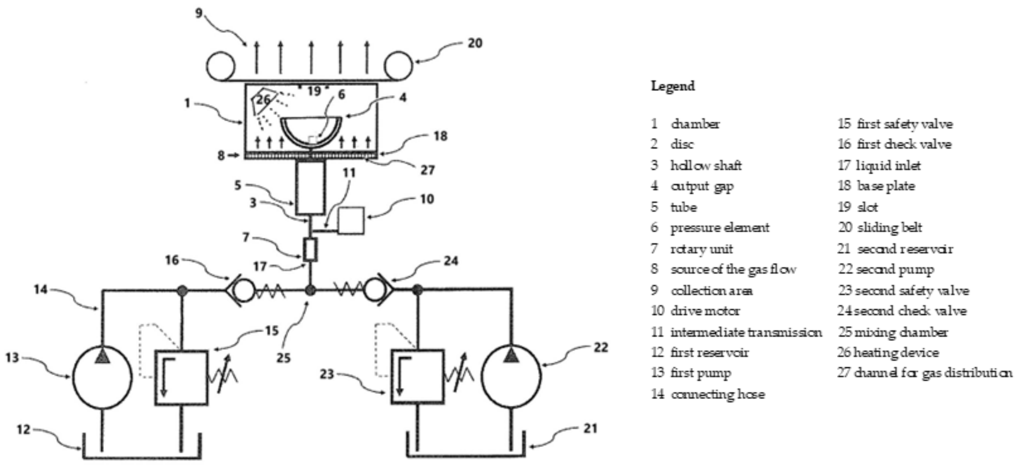

Figure S10. *Scheme of the demonstrator NANOCENT for nozzleless centrifugal spinning.*

We have not found any significant effect of the rottary speed of the spinning disk (rpm) on medians of the fiber diameter (µm). Medians of the fiber diameter range were in the range from 5,71 to 7,81 µm. All the samples of the 3D fiber constructs contained a mixture of submicron fibers and microfibers with very wide distibutions of the fiber diameter values. Histograms of PLA/PCL 13,5/4 fiber diameters obtained by the centrifugal spinning at different rotation speed are shown at **Figure S11**.

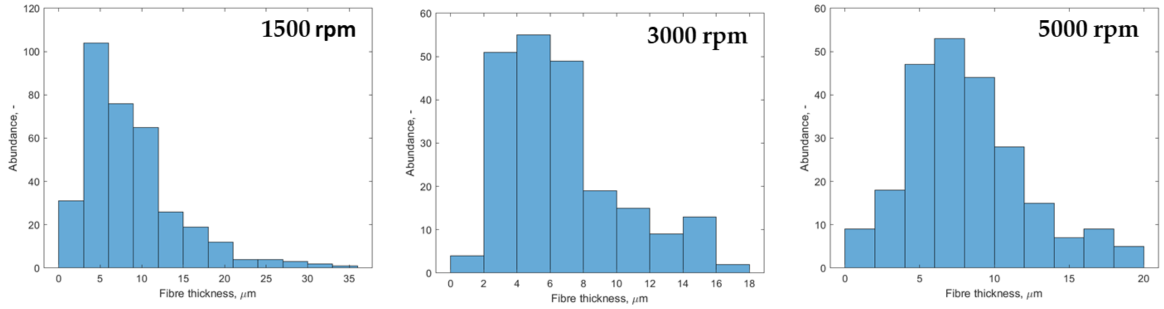

Figure S11. *Histograms of PLA/PCL 13,5/4 fiber diameters obtained by the centrifugal spinning at different rotation speed.*
